# Nanoparticles improve targeting and efficacy of endocytosis inhibitors

**DOI:** 10.1039/d6ra07148a

**Published:** 2026-10-22

**Authors:** Olga Klaudia Szewczyk-Roszczenko, Piotr Roszczenko, Iwona Misztalewska-Turkowicz, Rostyslav Dudchak, Ihor Yushyn, Yulia Shepeta, Serhii Holota, Anna Bielawska, Robert Czarnomysy, Roman Lesyk, Agnieszka Wilczewska, Yegor Vassetzky, Krzysztof Bielawski

**Affiliations:** a Department of Synthesis and Technology of Drugs, Medical University of Bialystok Kilinskiego 1 15-089 Bialystok Poland olga.szewczyk@sd.umb.edu.pl; b Department of Biotechnology, Medical University of Białystok Kilinskiego 1 15-089 Bialystok Poland; c Faculty of Chemistry, University of Bialystok Ciolkowskiego 1K Bialystok Poland; d Department of Pharmaceutical, Organic and Bioorganic Chemistry, Danylo Halytsky Lviv National Medical University Pekarska 69 79010 Lviv Ukraine; e Molecular Design Center, Danylo Halytsky Lviv National Medical University Pekarska 69 79010 Lviv Ukraine; f Department of Pharmaceutical Chemistry, National Pirogov Memorial Medical University Pirogova 56 21018 Vinnytsya Ukraine; g Department of Biotechnology and Cell Biology, Medical College, University of Information Technology and Management in Rzeszow Sucharskiego 2 35-225 Rzeszow Poland; h CNRS UMR9018 Institut Gustave Roussy, Université Paris Saclay 39 Rue Camille-Desmoulins 94805 Villejuif France

## Abstract

Endocytosis is a critical cellular process exploited by many viruses, including HIV-1 and SARS-CoV-2, for host cell entry. Inhibiting this pathway provides a promising antiviral strategy; however, existing small-molecule inhibitors often suffer from limited solubility, off-target effects, and poor bioavailability. This study reports the synthesis and evaluation of gold nanoparticles functionalized with novel endocytosis inhibitors, Les-6623 and Les-6632. The compounds were synthesized *via* a linear Knoevenagel condensation pathway and structurally confirmed by NMR and LC-MS spectroscopy. Molecular docking predicted more favorable interactions of Les-6623 and Les-6632 with dynamin-2 than those predicted for chlorpromazine, a classical endocytosis inhibitor. PEGylated gold nanoparticles were functionalized with the inhibitors, yielding formulations characterized by UV–Vis, fluorescence spectroscopy, ATR-FTIR, DLS, and TEM analyses. Functional assays in SH-SY5Y and BEAS-2B cells showed that AuNP_Les-6623 and AuNP_Les-6632 reduced the cellular fluorescence associated with transferrin, HIV-1 Tat, and SARS-CoV-2 Spike protein under the experimental conditions tested. Free chlorpromazine was included as a reference inhibitor. These findings support the biological activity of the investigated nanoparticle formulations under the tested conditions, while further format-matched controls are required to determine the specific contribution of nanoparticle presentation to the observed activity.

## Abstract 2

Endocytosis is a critical cellular process exploited by many viruses, including HIV-1 and SARS-CoV-2, for host cell entry.

## Graphical abstract

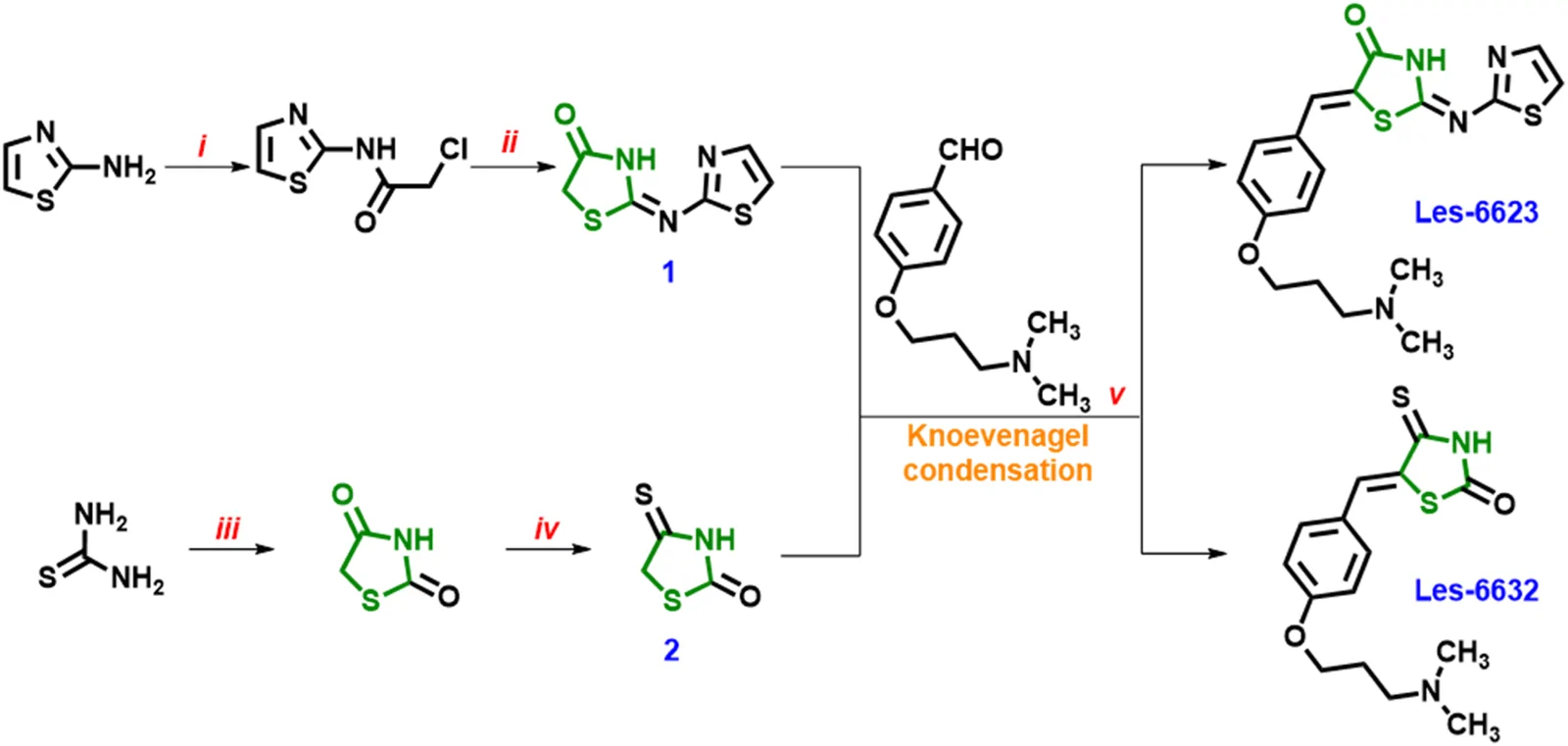

## 1. Introduction

Nanoparticles possess unique properties that make them particularly valuable in biomedical applications. Their relatively large surface area provides multiple binding sites for the attachment of diverse molecules, including therapeutic drugs, proteins, antibodies or targeting ligands, which enables the design of highly specialized delivery systems.^1^ Furthermore, nanoparticles can be synthesized in various shapes and sizes, such as spheres, rods, or shells, and these structural differences significantly influence their circulation time in the bloodstream, their interaction with biological barriers, and their ability to reach specific tissues or cellular targets.^2^ Another important feature is their capacity to release active substances in a controlled and sustained manner. This minimizes systemic toxicity and unwanted side effects by ensuring that the therapeutic agents are delivered in a localized and time-regulated fashion.^3^ As a result, nanoparticles are increasingly employed in diagnostics, imaging as well as in targeted drug delivery, gene therapy, immunomodulation, and the development of innovative therapeutic strategies.^4^

Gold nanoparticles are among the best-studied and most widely used nanomaterials in experimental medicine and molecular biology. They are characterized by high chemical stability, the possibility of straightforward synthesis in various sizes and shapes, and the ability to be efficiently functionalized. These features make them useful both as carriers of drugs and inhibitors, as well as in cellular imaging and photothermal therapy. From a safety perspective, gold in nanoparticle form exhibits relatively low toxicity and good biocompatibility, although their effects depend on dose, size, and surface modifications.^5^ Furthermore, growing attention has been directed toward functionalizing nanoparticles with biocompatible polymers or biological ligands to further enhance their stability, selectivity, and safety *in vivo*.^6,7^

Endocytosis plays a critical role in cellular communication with the surrounding environment. The most extensively characterized route is clathrin-mediated endocytosis, which involves the coordinated action of clathrin triskelions, adaptor protein complexes (*e.g.*, AP-2), and accessory proteins such as epsin and dynamin, the latter being responsible for vesicle scission. In contrast, caveolin-mediated endocytosis depends on caveolin-1 and cavin proteins, which stabilize cholesterol-rich membrane microdomains (caveolae) and regulate vesicle formation. Other pathways, including macropinocytosis, are driven by actin cytoskeleton remodeling and small GTPases such as Rac1 and Cdc42, leading to the formation of large, non-specific vesicles. Additionally, clathrin- and caveolin-independent pathways involve proteins such as flotillins, Arf6, and various lipid raft-associated components, reflecting a diverse set of internalization mechanisms. Importantly, these pathways differ not only in structure but also in cargo specificity, kinetics, and regulatory proteins, which necessitates careful experimental discrimination.^8^

Importantly, many pathogens, including viruses, exploit these pathways as an entry route into host cells. Viral proteins can interact with cell surface receptors and hijack endocytic machinery to promote viral uptake and replication. While essential for the viral life cycle, these proteins also represent a serious threat to the host by disrupting normal cellular functions. Their major roles include mediating viral entry, facilitating replication of the viral genome, and enabling evasion from host immune responses.^9^ For example, the Spike protein of SARS-CoV-2 specifically binds to the ACE2 receptor and activates endocytic processes, leading to efficient internalization of the virus into respiratory epithelial cells and beyond.^10^ The HIV-1 Tat protein is a regulatory factor that uses endocytosis to penetrate cells. Once internalized, Tat can interfere with transcriptional regulation, promote neurotoxicity, and contribute to the systemic pathogenesis of HIV infection.^11^ Beyond facilitating infection, viral proteins can drive chronic activation of the immune system, cause oxidative stress, and induce inflammatory responses that further exacerbate tissue injury and contribute to long-term complications associated with viral diseases.^12^ Blocking endocytosis is one way to prevent entry of viruses and viral proteins into cells.^8,13^ Because nanoparticles can alter the cellular association and presentation of surface-associated compounds, nanoparticle-based formulations may influence the biological activity of endocytosis inhibitors.^14^

The aim of this study was to develop nanoparticles conjugated with endocytosis inhibitors. As a proof of principle, we evaluated whether these nanoparticle-associated formulations reduce cellular cargo-associated fluorescence of the viral products (HIV-1 Tat and SARS-CoV-2 spike proteins) into cells. In this study, we evaluated whether gold nanoparticles functionalized with the endocytosis inhibitors Les-6623 and Les-6632 reduce the cellular uptake of transferrin, HIV-1 CPP Tat, and SARS-CoV-2 Spike protein under the experimental conditions used, with chlorpromazine included as a reference inhibitor.

## 2. Results and discussion

### 2.1. Synthesis of Les-6623 and Les-6632

The spectrum of available endocytosis inhibitors is limited, and many suffer from off-target effects. Pyrimidines, for instance, induce growth arrest in cancer cell lines,^15^ while Dynasore, Dynole, and Dyngo, though active against clathrin-dependent endocytosis, also interfere with receptor signaling, protein phosphorylation, and mTORC1 activation.^16,17^ Such nonspecific activities preclude their clinical use. To address these shortcomings, we previously developed rhodadyn-based inhibitors Les-6631 and Les-6633 and compared them with chlorpromazine.^18^ Rhodanine scaffolds selectively target dynamin II GTPase activity without impairing clathrin, even at higher concentrations.^19^ Consistently, Les-6631 and Les-6633 blocked the uptake of Tat-Cy5 CPP and dextran in SH-SY5Y and MCF-7 cell lines.^13^ However, their application was constrained by limited solubility, which reduced their practical utility despite promising selectivity, which inspired us to project a safe and sustainable delivery system.

A linear synthetic scheme was used for the synthesis of the 4-thiazolidinone-based derivatives Les-6623 and Les-6632 as non-salt analogues of early reported endocytosis inhibitors Les-6623 and Les-6632 (Fig. 1). Key scaffolds 1 and 2 were obtained following the protocols.^20,21^ The starting building block, 2-(thiazol-2-ylimino)thiazolidin-4-one,^1^ was synthesized from 2-aminothiazole by initial reaction with chloroacetyl chloride to yield the corresponding chloroacetamide, followed by cyclization with ammonium thiocyanate. 4-Thioxo-2-thiazolidinone (isorhodanine, 2) was obtained from thiourea *via* heterocyclization with chloroacetic acid, followed by thionation of the C-4 carbonyl group using phosphorus pentasulfide. Synthesized compounds were purified by recrystallisation from ethanol^1^ and water.^2^ In the next stage, for the synthesis of target inhibitors, Knoevenagel condensation was applied. Derivatives Les-6623 and Les-6632 were synthesized in the reaction of compounds 1 or 2 with 4-(3-(dimethylamino)propoxy)benzaldehyde with presence of EDDA catalyst in ethanol by reflux. Molecules Les-6623 and Les-6632 were obtained with satisfactory yield 70–90% and additionally purified by recrystallisation from mixture DMF : EtOH.

**Fig. 1:**
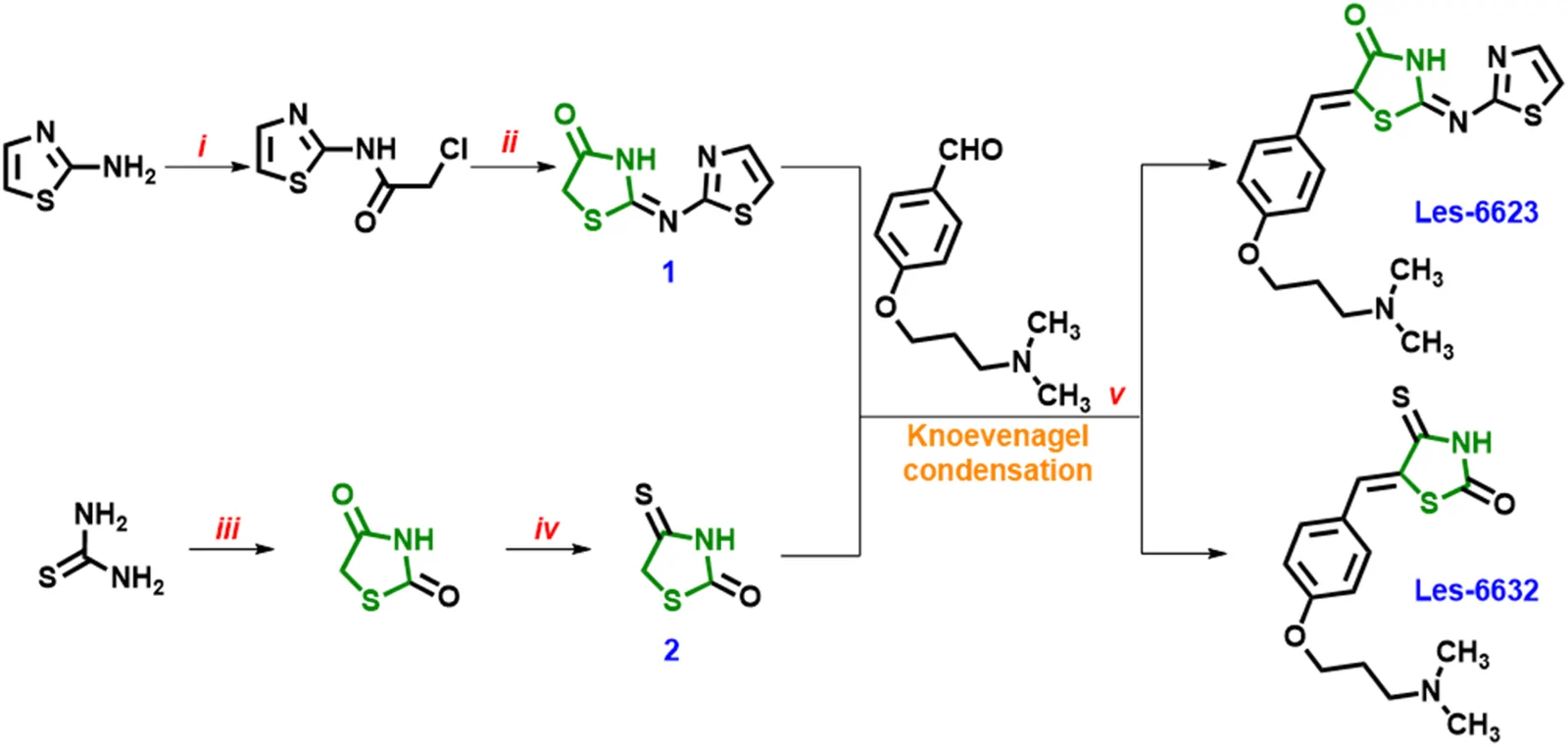
Synthetic route to the 4-thiazolidinone-based derivatives Les-6623 and Les-6632. Reagents and conditions: (i) thiazol-2-amine (10 mmol), chloroacethyl chloride (10 mmol), triethylamine (10 mmol), dioxane (10 mL), r.t. 2 h; (ii) 2-chloro-*N*-(thiazol-2-yl)acetamide (10 mmol), NH_4_SCN (20 mmol), acetone (10 mL), reflux 5 h; (iii) thiourea (10 mmol), chloroacetic acid (10 mmol), HCl (10 mL), reflux 3 h; (iv) thiazolidine-2,4-dione (10 mmol), P_2_S_5_ (10 mmol), dioxane (10 mL), r.t. 5 h; (v) 2-(thiazol-2-ylimino)thiazolidin-4-one (1) or 4-thioxo-2-thiazolidinone (isorhodanine) (2) (10 mmol), 4-(3-(dimethylamino)propoxy)benzaldehyde (11 mmol), EDDA (10 µmol), EtOH (10 mL), reflux, 2 h (for comp. 1) or 10 min (for comp. 2), 70–90%.

The structure of the synthesized compounds Les-6623 and Les-6632 was confirmed by ^1^H, ^13^C NMR and LC-MS spectra (copies of spectra are presented in the SI). In the ^1^H NMR spectra of compound Les-6623, the ylidene proton resonates as a singlet at 7.89 ppm, whereas for Les-6632, this signal overlaps with the aromatic proton multiplet. The aliphatic protons of the 3-(dimethylamino)propoxy moiety for both Les-6623 and Les-6632 derivatives exhibit a characteristic signal pattern with appropriate spin–spin coupling constants in the region of 2.00–4.10 ppm. In the ^13^C NMR spectra, the signals of the carbonyl carbon (C=O) appear at 163.50 ppm (Les-6623) and 170.40 ppm (Les-6632), while the thiocarbonyl carbon (C=S) signal resonates at 192.2 ppm. The molecular ion peaks observed at the *m*/*z* value of 323 (Les-6623) and 389 (Les-6632) [M + H]^+^ in positive ionization mode in the mass spectrum confirmed obtaining of target compounds.

### 2.2 Molecular docking of interactions with dynamin-2

To evaluate the potential interactions of the synthesized compounds with dynamin-2, molecular docking studies were performed, as this approach is widely used for the *in silico* prediction of ligand–protein interactions. Prior to docking, potential ligand-binding pockets were identified using the SiteMap function of Schrödinger Suite. As shown in Table 1, five potential binding sites were predicted on the surface of dynamin-2. SiteMap ranks binding pockets using two parameters: Site Score, which reflects the overall suitability of a binding site based on its geometric and physicochemical properties, and Drug Score, which estimates its “druggability”. Binding sites with a Site Score > 1.0 are considered promising, whereas Drug Score values > 0.98 indicate “druggable” pockets, values between 0.83 and 0.98 indicate moderately “druggable” sites, and values < 0.83 suggest low “druggability”. Among the identified pockets, Site 2 fulfilled both selection criteria, exhibiting a Site Score of 1.030 and a Drug Score of 1.047. These results indicate that Site 2 represents the most suitable binding pocket for ligand binding and was therefore selected for subsequent molecular docking studies. Although Site 1 achieved the highest Site Score (1.082), its Drug Score of 0.815 suggests limited “druggability” compared with Site 2. Accordingly, a docking grid was generated for Site 2 and used for all subsequent molecular docking calculations (Fig. 2).

**Table 1:** Site score and drug score values for the predicted binding pockets of dynamin-2 identified by SiteMap

Site	1	2	3	4	5
Site score	1.082	1.030	0.996	0.993	0.980
Drug score	0.815	1.047	1.029	1.026	0.980

**Fig. 2:**
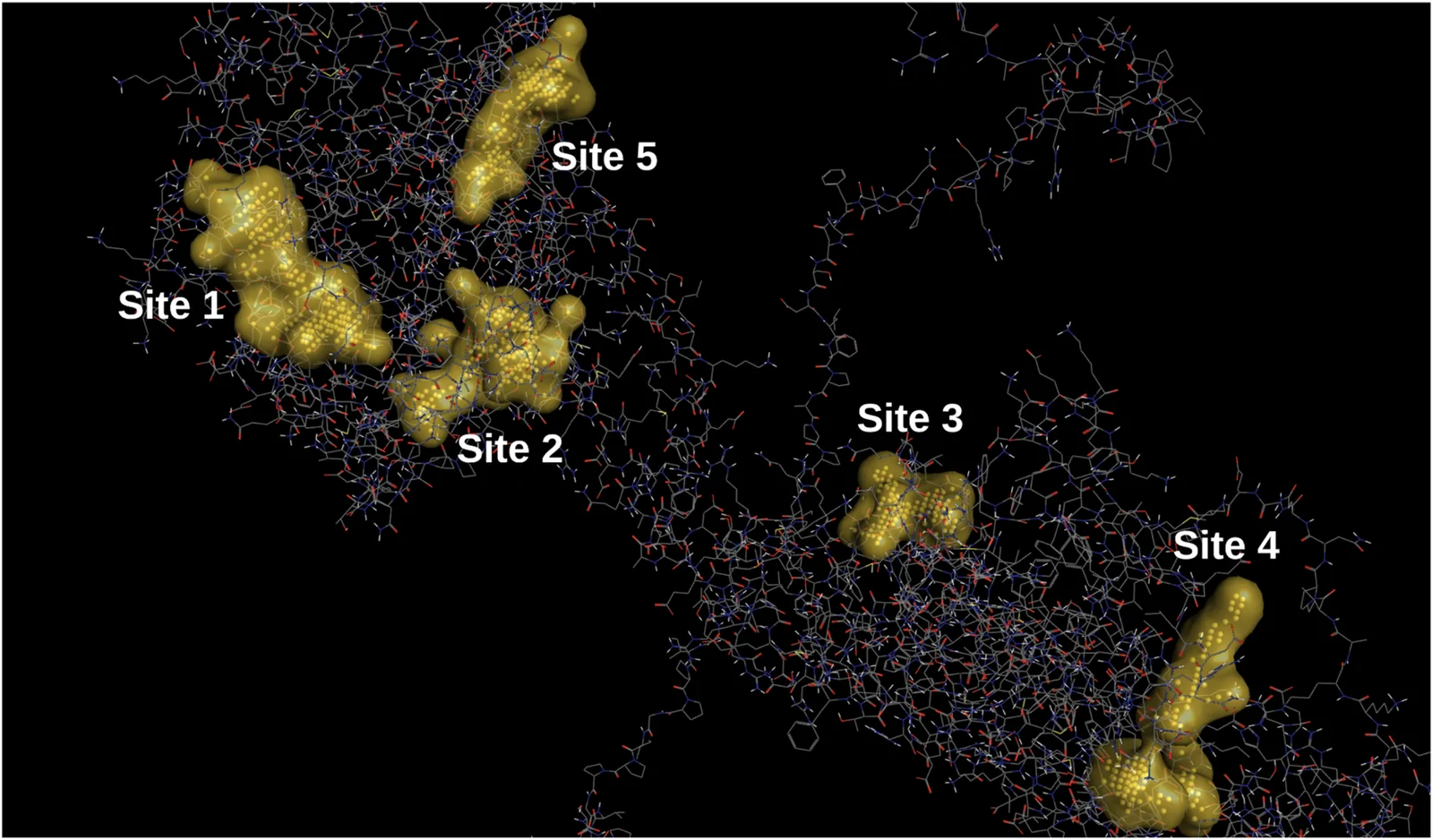
Predicted ligand-binding pockets on the surface of dynamin-2 identified using the SiteMap module of the Schrödinger Suite (release 2024-4).

Molecular docking was performed to assess the interaction of the synthesized compounds with the selected binding pocket of dynamin-2. As presented in Table 2, both Les-6623 and Les-6632 yielded more favorable docking scores than chlorpromazine. Since lower docking scores indicate more favorable ligand–protein interactions. These results suggest a stronger predicted binding of both compounds to dynamin-2. Les-6623 showed the lowest docking score (−6.620 kcal mol^−1^), indicating the most favorable binding among the tested compounds within the selected docking model. Furthermore, both Les compounds exhibited better ligand efficiency than chlorpromazine, suggesting more efficient binding relative to their molecular size.

**Table 2:** Molecular docking scores (kcal mol^−1^) and ligand efficiency values for Les-6623, Les-6632, and chlorpromazine docked to the selected binding pocket of dynamin-2. Molecular docking results for tested compound and chlorpromazine displayed in kcal mol^−1^

Name	Docking score	Ligand efficiency
Les-6623	−6.620	−0.315
Les-6632	−6.172	−0.237
Chlorpromazine	−5.800	−0.276

### 2.3. Cytotoxicity of compounds Les-6623 and Les-6632

Cytotoxicity assessment of the synthesized compounds Les-6623 and Les-6632 was performed using the MTT assay on a panel of six cell lines, including A172 and U87 (glioblastoma), A549 (lung adenocarcinoma), Panc-1 (pancreatic carcinoma), SH-SY5Y (neuroblastoma), and the non-malignant BEAS-2B bronchial epithelial cell line. As summarized in Table 3, neither Les-6623 nor Les-6632 exhibited measurable cytotoxic effects after 48 h of incubation in any of the tested cell lines, with IC_50_ values exceeding 100 µM in all cases. The obtained results demonstrate that both compounds exhibited no measurable cytotoxicity in any of the tested cell lines under the applied experimental conditions.

**Table 3:** Summary of cytotoxicity (IC_50_, µM) of Les-6623, Les-6632 and chlorpromazine in human cell lines after 48 h of incubation

Compound	Les-6623	Les-6632	Chlorpromazine
A172	>100	>100	38.5
A549	>100	>100	15.0
BEAS-2B	>100	>100	22.7
Panc-1	>100	>100	23.7
SH-SY5Y	>100	>100	18.5
U87	>100	>100	32.5

### 2.4. Functionalization and characterization of nanoparticles

One of the most common strategies to improve the stability and safety of gold nanoparticles is coating them with PEG. PEGylation prevents particle aggregation, prolongs their circulation in the bloodstream, and reduces recognition by the immune system, thereby limiting the risk of rapid clearance. With these properties, PEG-coated gold nanoparticles represent an attractive platform for the development of novel therapeutic systems.^22^ This was our reasoning behind choosing a specific model to connect compounds Les-6623 and Les-6632 with gold nanoparticles.

#### 2.4.1 Ultraviolet–visible (UV–Vis) absorption spectroscopy

UV–Vis absorption spectra were recorded for gold nanoparticles coated with a PEG layer and further modified with compounds Les-6623 and Les-6632. The PEG-coated sample exhibited a maximum absorption at 523 nm. The addition of Les-6623 and Les-6632 resulted in a shift of the maximum towards shorter wavelengths by 3 nm and 2 nm, respectively, AuNPs_Les-6623 showed a maximum at 520 nm, while AuNPs_Les-6632 exhibited a maximum at 521 nm. This change is within the range typically associated with minor changes in the nanoparticle surface environment. In the spectrum of AuNPs_Les-6632, an additional maximum was observed at 384 nm, consistent with an absorption band of Les-6632. In contrast, the spectrum of AuNPs_Les-6623 did not display distinct absorption bands attributable to the modifying compound (Fig. S7 and S8).

#### 2.4.2 Fluorescence spectroscopy

Gold nanoparticles coated with a PEG layer exhibited fluorescence when excited with a wavelength of 520 nm, with the emission maximum observed at 582 nm. The compounds used for nanoparticle modification, Les-6623 and Les-6632, displayed fluorescence under excitation at 345 nm and 286 nm, respectively, showing emission maxima at 471 nm (Les-6623) and 323 nm (Les-6632). Gold nanoparticles modified with these two drugs no longer exhibited fluorescence when illuminated at 520 nm, consistent with interaction with the particles, which quenched their fluorescence. Furthermore, no fluorescence was detected when the modified nanoparticles were excited at the wavelengths that induced fluorescence in the pure compounds, *i.e.*, Les-6623 and Les-6632. In the case of AuNPs_Les-6623, fluorescence was observed upon excitation at 275 nm, while excitation of AuNPs_Les-6632 with a 385 nm wavelength led to a weak fluorescence signal with a maximum at 443 nm. The changes in the fluorescence spectra are consistent with surface modification (Fig. S10 and S11).

#### 2.4.3 Attenuated total reflectance Fourier transform infrared (ATR-FTIR)

In the spectrum of PEG-coated gold nanoparticles, distinct signals originating from the polymer shell were observed: C–O stretching vibrations (1111 cm^−1^) from the polymer chain, as well as C=O stretching vibrations corresponding to the carboxyl group (1738 cm^−1^) and carboxylate salt (1636 cm^−1^). The symmetric and asymmetric C–H stretching bands were overlapped by a very intense peak at 2871 cm^−1^, most likely arising from hydrogen bonding interactions between carboxyl groups. This peak disappeared after the addition of compounds Les-6623 and Les-6632, supporting surface modification of the nanoparticles. In the spectra of AuNPs_Les-6623 and AuNPs_Les-6632, characteristic peaks corresponding to aromatic C–H vibrations (∼3000 cm^−1^) and aliphatic C–H vibrations (∼2918 cm^−1^) were observed. Additionally, a shift of the C–O stretching vibration band from the PEG chain by 102 cm^−1^ and 105 cm^−1^ (for AuNPs_Les-6623 and AuNPs_Les-6632, respectively) was detected. A disappearance in the band assigned to the free carboxyl group (1738 cm^−1^) was observed, accompanied by the appearance of a more intense band at approximately 1650 cm^−1^. These spectral changes are consistent with an altered ionic environment of the carboxyl groups following interaction with the protonated amine groups of Les-6623 and Les-6632. The formation of the salt was further supported by the appearance of a broad and intense band around 3400 cm^−1^, corresponding to NH^+^ groups (Fig. 3). The FTIR data therefore support the proposed surface association of the compounds with the carboxyl-containing PEG layer, but do not provide a quantitative determination of the amount of compound associated with the nanoparticles.

**Fig. 3:**
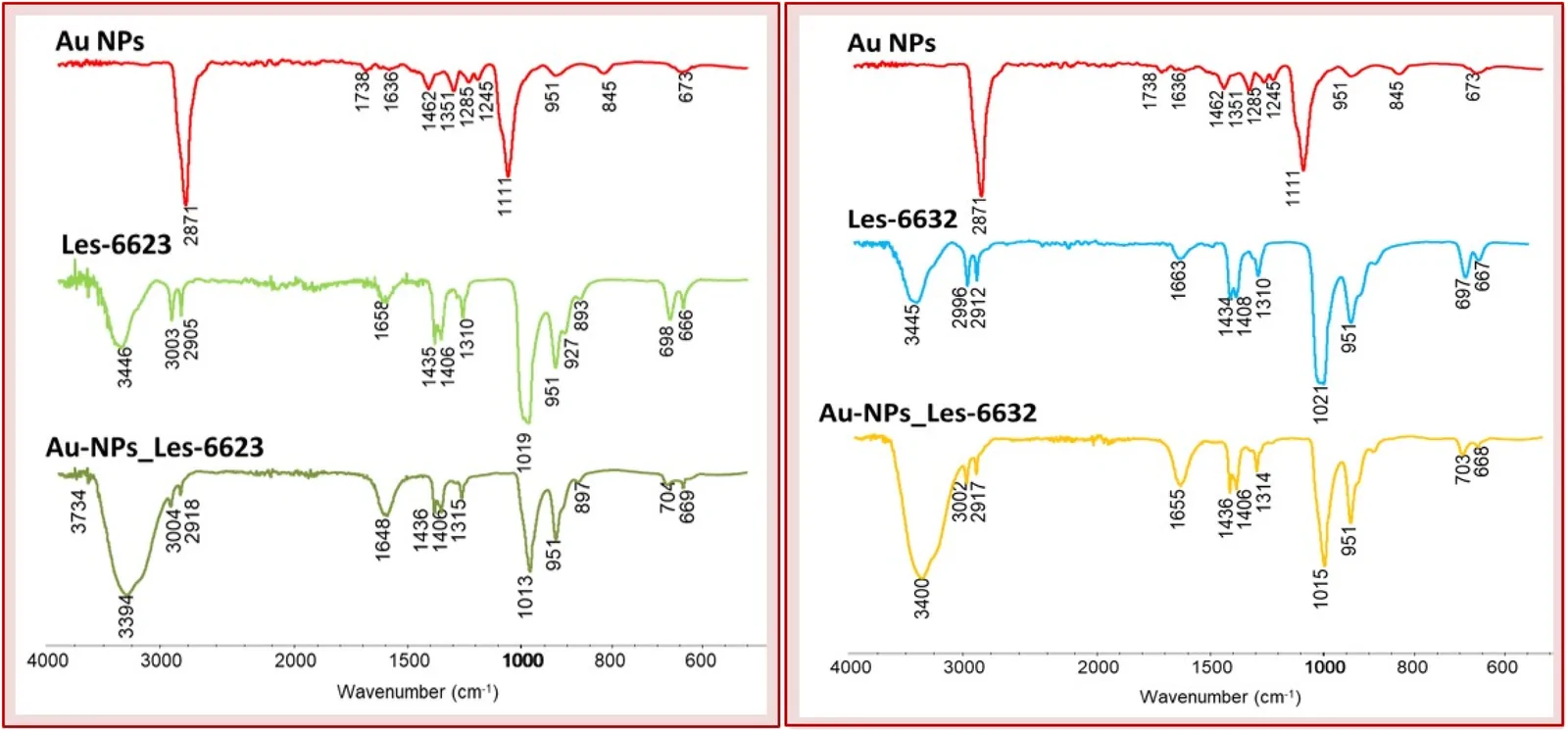
FT-IR spectra of AuNPs_Les-6623 and AuNPs_Les-6632 particles in comparison with the initial compounds.

#### 2.4.4 Dynamic light scattering (DLS)

PEG-coated AuNPs were analyzed by DLS. Their size (hydrodynamic diameter) ranged from 9 to 42 nm. PEG-modified AuNPs exhibited a zeta potential value of −8.306 (average of 15 measurements). Upon the addition of compound Les-6623, the zeta potential initially decreased to nearly zero (−0.7657) and subsequently stabilized at a value comparable to that of the unmodified nanoparticles (−8.303). This rapid change may indicate self-assembly processes occurring during the measurement, likely induced by the applied electric field. The subsequent stabilization of the zeta potential suggests that an equilibrium state was reached. Analysis of particles modified with compound Les-6632 revealed a decrease in zeta potential to −22.95. Following the addition of both compounds, no aggregation of the gold nanoparticles into larger clusters was observed, and the particle size remained within the range of 13–44 nm. The stability of the nanoparticles was assessed 48 hours and 8 days after drug addition, and no significant changes were detected in either zeta potential or particle size (Fig. S12). These measurements indicate colloidal stability under the tested storage conditions but do not directly demonstrate the presence or integrity of the PEG coating.

#### 2.4.5 Transmission electron microscopy (TEM)

The structure and size of the nanoparticles were examined using TEM. TEM images confirmed that the size of AuNPs core was within the range of 8–12 nm, consistent with the manufacturer's specifications. Nanoparticles modified with Les-6623 and Les-6632 exhibited the shape and size similar to those of the initial PEG-coated particles, although the PEG layer cannot be directly visualized. Small aggregates composed of two or three nanoparticles were observed, resulting in larger particles of irregular shape. Conventional TEM visualizes only the metallic core; therefore, conclusions are limited to preservation of core morphology (Fig. 4).

**Fig. 4:**
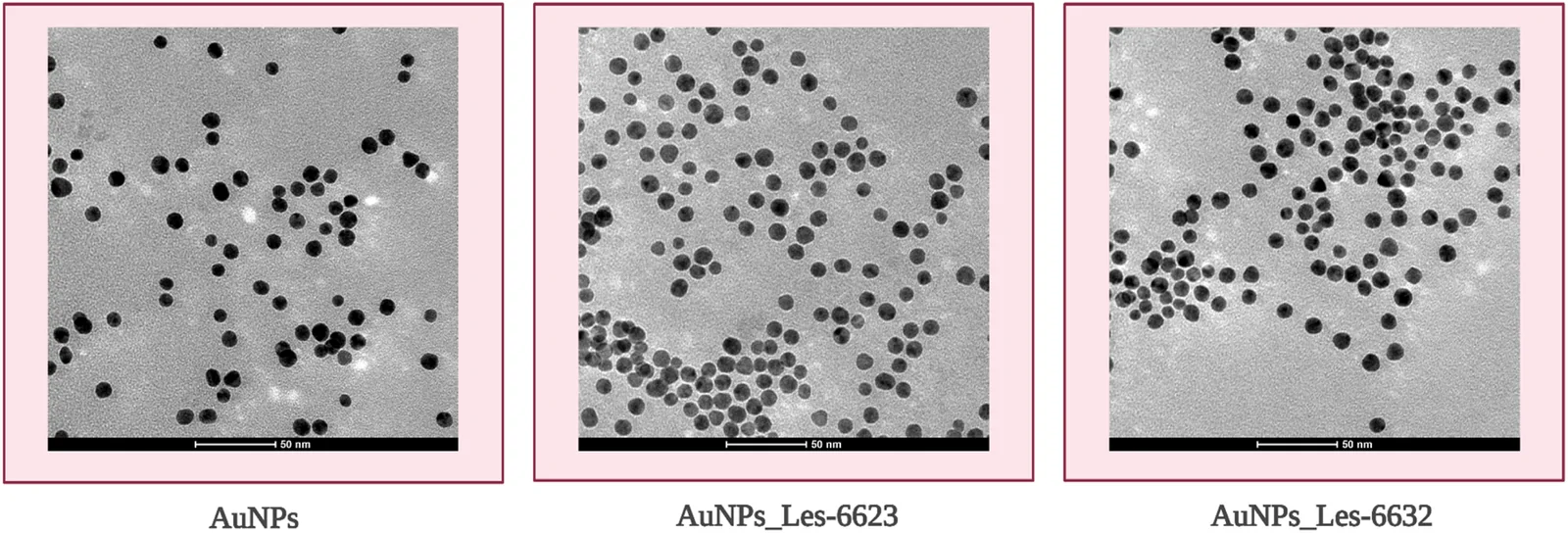
Representative TEM images of AuNPs, AuNPs_Les-6623 and AuNPs_Les-6632. Scale bar = 50 nm.

### 2.5. Comparative analysis of transferrin endocytosis inhibition by AuNPs_Les-6623 and AuNPs_Les-6632

Transferrin is typical cargo transported by endocytosis inside the cells.^23^ We evaluated the effect of nanoparticle-conjugated Les-6623 and Les-6632 in SH-SY5Y cells. The intensity of transferrin labeled with Alexa Fluor 647 was quantified by measuring fluorescence. Fig. 5A represents a quantitative analysis of transferrin uptake under different experimental conditions, measured as described in Materials and Methods by confocal microscope. Control cells exhibited the highest fluorescence level. Treatment with 30 µM chlorpromazine resulted in a decrease in fluorescence, consistent with its well-documented role as an endocytosis inhibitor. The inhibitory effect observed for chlorpromazine was lower than reported in some studies, possibly because uptake experiments were performed in a complete medium containing serum.^24^ A significant difference in transferrin-associated fluorescence was observed between the nanoparticle formulations and the corresponding free compounds at a nominal concentration of 10 µM. For Les-6623, the nanoparticle formulation showed 19.23 ± 5.57% lower fluorescence than the corresponding free compound under the tested conditions, whereas for Les-6632, fluorescence was reduced by 34.51 ± 5.76%. These results indicate a greater reduction in transferrin-associated cellular fluorescence for the nanoparticle formulations than for the corresponding free compounds under the experimental conditions tested.

**Fig. 5:**
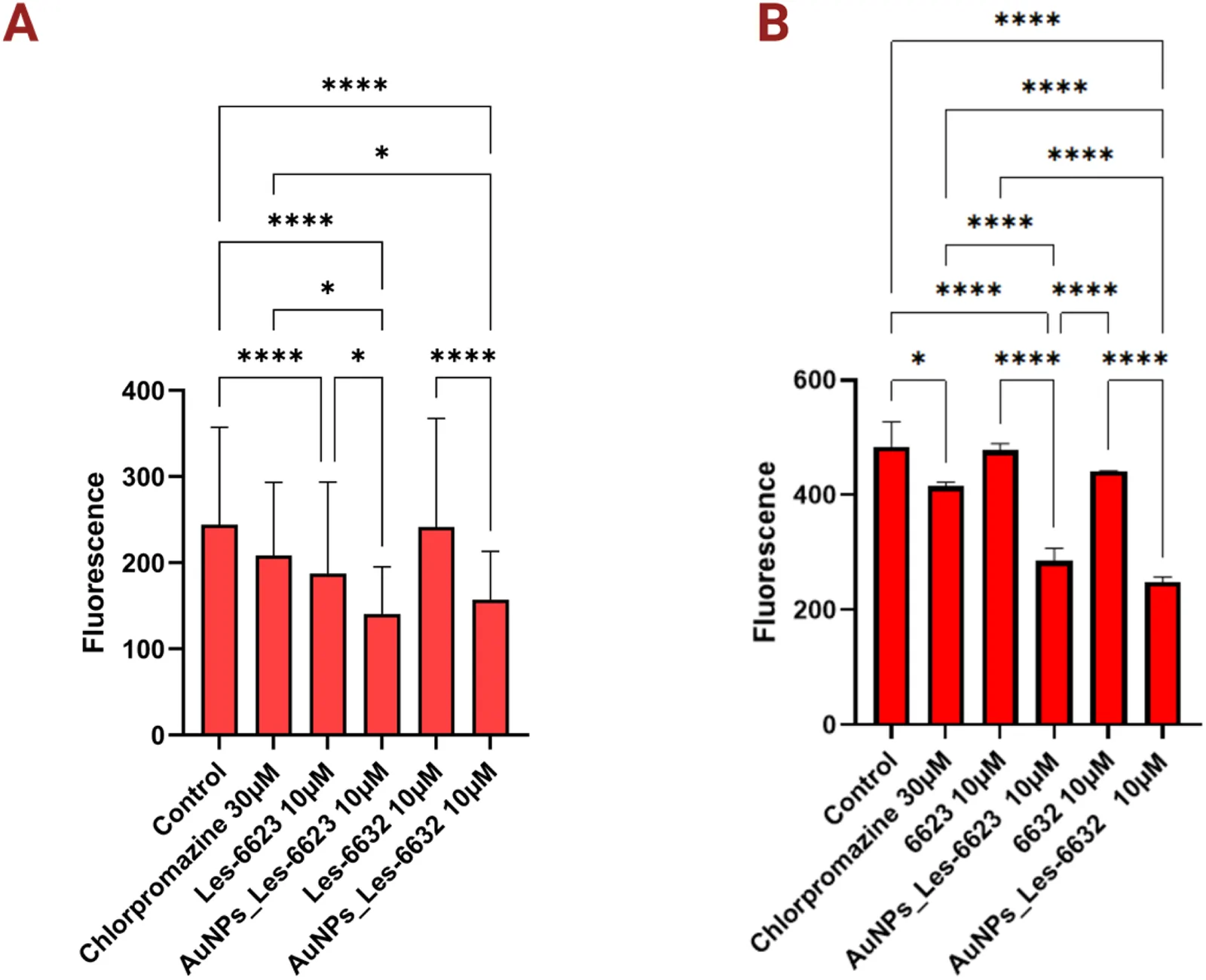
Effects of AuNPs_Les-6623 and AuNPs_Les-6632 on transferrin-associated cellular fluorescence in SH-SY5Y cells. Nanoparticle formulations were prepared using 10 µM nominal Les-6623 or Les-6632, respectively. Histogram A: the fluorescence intensity of AuNPs_Les-6623 and AuNPs_Les-6632 inhibition analyzed by confocal microscope, means ± SD are shown with *n* = 100; Histogram B: the fluorescence intensity of cargo entry analyzed by FACS, means ± SD are shown with *n* = 3, (20 000 events each). ****P ≤ 0.0001; **P ≤ 0.01; *P ≤ 0.05.

Flow cytometry was performed as an independent quantitative method to validate the confocal microscopy observations at the whole-cell population level. Fig. 5B presents a quantitative analysis of transferrin uptake in SH-SY5Y cells under the same experimental conditions, but with FACS analysis on living cells. As in panel A, control cells exhibited the highest fluorescence levels. Treatment with 30 µM chlorpromazine reduced transferrin fluorescence intensity, suggesting its inhibitory effect on endocytosis. AuNPs_Les-6623 and AuNPs_Les-6632 also led to a statistically significant decrease in fluorescence compared to control.

Statistical analysis confirmed differences both between the control and all experimental groups as well as between the free compounds and their nanoparticle formulations (*p < 0.05; ****p < 0.0001), indicating a greater reduction in transferrin-associated fluorescence for the nanoparticle formulations than for the corresponding free compounds under the tested conditions. Unfunctionalized AuNP-PEG controls were not included in these experiments; therefore, the contribution of the nanoparticle carrier itself to the observed reduction in transferrin-associated fluorescence cannot be excluded.

### 2.6. Inhibition of Tat CPP endocytosis

HIV-1 Tat protein is able to enter cells due to its cell-penetrating peptide (CPP^25,26^). While Tat-derived peptides are often described as being internalised mainly *via* macropinocytosis, accumulating evidence indicates that CPP uptake is not restricted to a single pathway but can involve multiple endocytic mechanisms. In particular, several studies have shown that dynamin-dependent processes may contribute to the internalisation of Tat constructs, supporting the use of pTat-Cy5 as a relevant, but not exclusive, model for assessing inhibition of dynamin-mediated uptake.^27,28^

Internalization of Cy5-labeled Tat CPP was quantified by measuring cell-associated fluorescence. Cells were pretreated for one hour with different concentrations of nanoparticle-conjugated Les-6623, Les-6632 or chlorpromazine, while untreated cells served as controls. Following pretreatment, Tat CPP-Cy5 was added to the full culture medium with 10% serum and incubated for 30 minutes. After incubation, the cells were washed thoroughly, fixed with 4% paraformaldehyde, and examined microscopically (Fig. 6A).

**Fig. 6:**
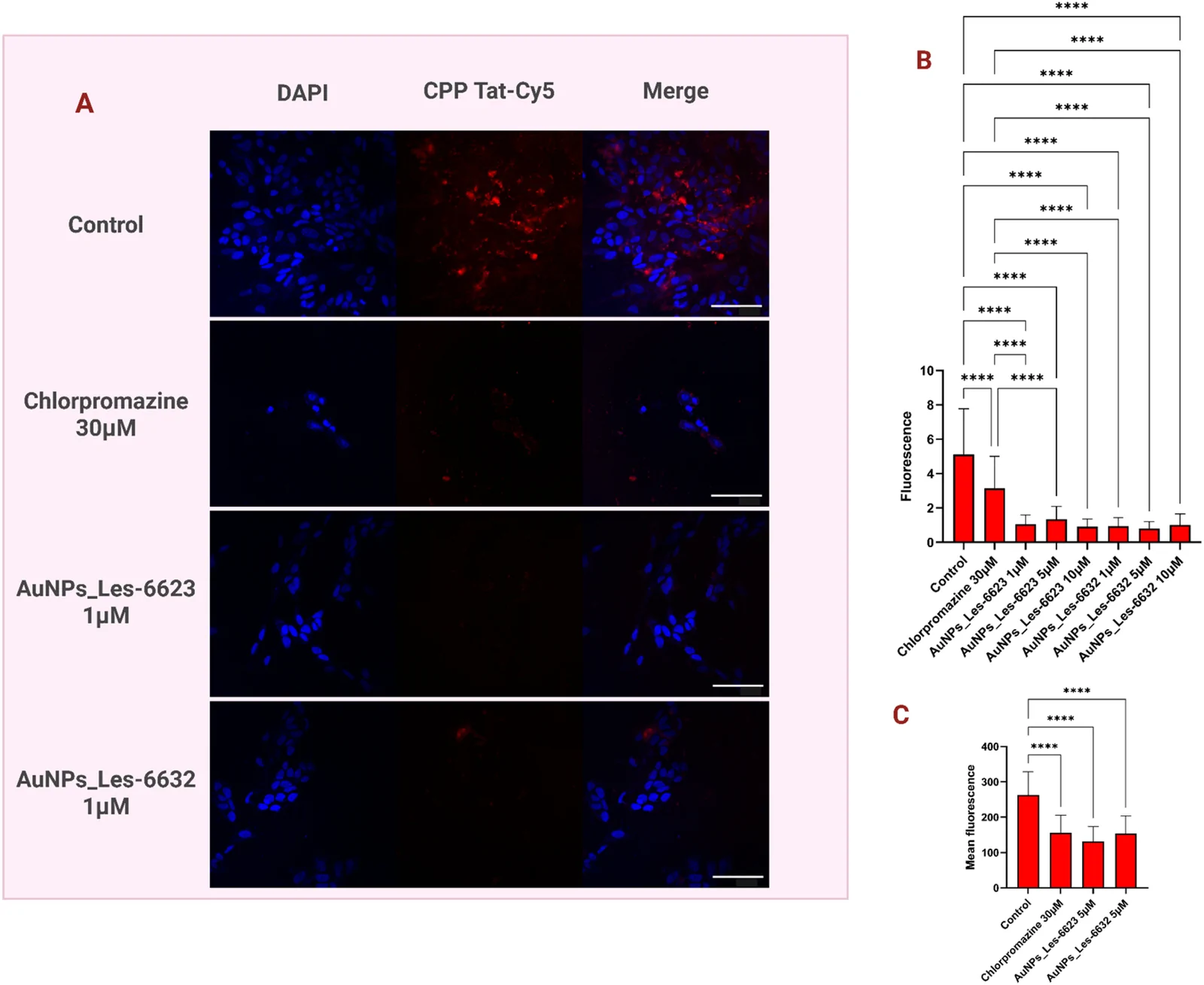
AuNPs_Les-6623 and AuNPs_Les-6632 suppress cellular uptake of fluorescently-labelled proteins into SH-SY5Y and BEAS-2B cell lines. A: representative confocal microscope images of the SH-SY5Y cells pretreated for 1 h either with the reference compound chlorpromazine at a concentration of 30 µM, or the Les compounds compared to AuNPs_Les-6623 and AuNPs_Les-6632 prior to CPP Tat cargo addition. Then, cargo was added to the cell medium for 30 min. Following this, the cells were thoroughly washed and stained with DAPI to visualize cell nuclei. Scale bar = 50 µm Histogram B: fluorescence intensity associated with CPP HIV-1 Tat in SH-SY5Y cells; Histogram C: fluorescence intensity associated with SARS-CoV-2 Spike protein in BEAS-2B cells. Means ± SD are shown with *n* = 100. ANOVA tests demonstrated statistically significant differences ****P ≤ 0.0001; *P ≤ 0.05.

Fig. 6B represents a histogram illustrating the fluorescence levels of cells under different experimental conditions. The control sample showed the highest fluorescence intensity of CPP Tat-Cy5 into the cells. Treatment with chlorpromazine at 30 µM markedly reduced fluorescence (61.53 ± 6.78% of the control), suggesting inhibition of endocytosis. A strong reduction was observed for gold nanoparticles modified with Les-6623 and Les-6632, where fluorescence intensity was significantly lower compared to the control (17.68 ± 0.64% and 19.88 ± 0.68% of control at a nominal concentration of 10 µM, respectively). The decrease in fluorescence was observed across the tested concentrations of the nanoparticle formulations. Under the experimental conditions used, the nanoparticle formulations produced lower Tat-associated fluorescence than the chlorpromazine reference treatment. Unfunctionalized AuNP-PEG controls were not included in these experiments. Statistical analysis demonstrated high significance (****, *p* < 0.0001).

A similar trend was observed for CPP Tat-Cy5 in our previous manuscript. We showed that 10 µM concentration of compounds did not statistically differ from chlorpromazine (Szewczyk-Roszczenko *et al.*, 2025; Fig. 3 compounds Les-6623 and Les-6632 are non-salt versions of Les-6631 and Les-6633 respectively).^13^

### 2.7. Assessment of SARS-CoV-2 spike protein inhibition

Additionally to CPP Tat, we tested another viral protein, SARS-CoV-2 spike that normally enters cells by endocytosis.^29^ The bar graph (Fig. 6C) shows the mean fluorescence intensity of cells under treatment with different compounds. In the control sample, the fluorescence signal was the highest, indicating efficient uptake of the fluorescently labeled SARS-CoV-2 spike. Treatment with chlorpromazine at 30 µM significantly reduced the fluorescence level (59.14 ± 2.94% of control). A comparable decrease in fluorescence was also observed for nanoparticles AuNPs_Les-6623 (nominal 5 µM) and AuNPs_Les-6632 (nominal 5 µM) 49.81 ± 2.24% and 58.36 ± 2.49% of control, respectively. Statistical analysis demonstrated high significance (****, *p* < 0.0001) for all treatment groups compared to control, confirming that both chlorpromazine and the functionalized nanoparticles effectively reduce spike-associated fluorescence of the SARS-CoV-2 spike. Unfunctionalized AuNP-PEG controls were not included in these experiments; therefore, the contribution of the nanoparticle carrier itself to the observed reduction in transferrin-associated fluorescence cannot be excluded.

Overall, our study shows that gold nanoparticles functionalized with the 4-thiazolidinone derivatives Les-6623 and Les-6632 reduce the cellular fluorescence associated with HIV-1 Tat and SARS-CoV-2 Spike protein under the experimental conditions tested. The nanoparticle formulations exhibited colloidal stability under the tested conditions and low measurable cytotoxicity of the investigated compounds, while differences in cargo-associated fluorescence were observed relative to the corresponding free compounds and the chlorpromazine reference treatment. These findings suggest that nanoparticle association may improve inhibitory activity under the tested conditions. However, dynamin itself represents a highly pleiotropic and context-dependent target involved in multiple cellular processes beyond endocytosis, which may constrain the specificity and translational applicability of its inhibition^30^

### 2.8. Limitations of the study

'The present study has several limitations that should be considered when interpreting the results. First, although nanoparticle functionalization was supported by complementary physicochemical characterization (ATR-FTIR, UV–Vis spectroscopy, fluorescence spectroscopy, DLS and TEM), the actual amount of Les-6623 and Les-6632 associated with the PEG-coated gold nanoparticles was not quantitatively determined. The stoichiometric amount of compound introduced during formulation was calculated from the concentration of accessible surface carboxyl groups determined by pH-metric titration; however, this represents the nominal amount introduced rather than a direct measurement of nanoparticle-associated drug. ATR-FTIR supports the proposed ionic association but cannot provide a quantitative mass balance of bound and unbound compounds. Therefore, all compound concentrations reported for the nanoparticle formulations should be understood as nominal concentrations based on the amount introduced during formulation, and the biological dose–response relationships should be interpreted accordingly.

Second, the biological activity of PEG-coated gold nanoparticles without conjugated inhibitors was not evaluated, because of overlap of fluorescence between cargo and non-functionalized nanoparticles. Therefore, although the observed effects are consistent with the activity of nanoparticle-associated Les compounds, a potential contribution of the nanoparticle carrier itself cannot be completely excluded in case of HIV-1 Tat and SARS-CoV-2 Spike. Additionally the experiments were performed in complete culture medium containing 10% fetal bovine serum, whereas chlorpromazine is commonly evaluated under serum-free conditions to minimize interactions with serum components. Consequently, the inhibitory effect of chlorpromazine observed in the present study may have been influenced by the presence of serum, which could contribute to the lower apparent inhibition of transferrin uptake compared with values reported in the literature.

Another important limitation concerns the comparison with free chlorpromazine. Chlorpromazine was included as a reference inhibitor under the same experimental conditions; however, this comparison is not format-matched. The investigated compounds and chlorpromazine differ in chemical structure, and the investigated compounds were additionally presented in a nanoparticle-associated format. Therefore, the present experimental design cannot distinguish the contribution of inhibitor structure from that of nanoparticle presentation to the observed differences in activity. A format-matched comparison, such as chlorpromazine-functionalized nanoparticles *versus* the investigated nanoparticle formulations, would be required to determine whether the nanoparticle format itself contributes to the observed difference. Accordingly, the present results should not be interpreted as demonstrating that the nanoformulation intrinsically outperforms free chlorpromazine because of the nanoparticle delivery format.

''Third, the uptake assays used in this work were designed as comparative measurements of cellular cargo association under identical experimental conditions. These methods do not unambiguously distinguish between surface-bound and fully internalized cargo, nor do they discriminate between cargo retained in endocytic intermediates and cargo delivered to intracellular compartments. Consequently, the observed reduction in fluorescence should be interpreted as reduced cellular cargo uptake under the applied experimental conditions rather than as a direct quantitative measurement of endocytic flux.

Furthermore, although transferrin and Tat peptides are widely used as model cargoes in studies of endocytosis, their internalization may involve multiple uptake pathways depending on cell type and experimental conditions. Therefore, the present experiments do not establish the exclusive involvement of dynamin-dependent endocytosis.

Finally, the present work was designed as a proof-of-concept study demonstrating that nanoparticle association enhances the biological activity of previously identified endocytosis inhibitors. Direct biochemical confirmation of dynamin inhibition, detailed pathway-specific analyses, serum stability studies, protein corona characterization, and *in vivo* evaluation remain important subjects for future investigation.

Taken together, the physicochemical and biological data support the biological activity of the investigated nanoparticle formulations under the experimental conditions used, while the quantitative drug loading, the contribution of nanoparticle presentation, and the specific endocytic mechanisms underlying the observed effects remain to be established.

## 3. Materials and methods

### 3.1. General procedure for the synthesis of 5-(4-(3-(dimethylamino)propoxy)benzylidene)-thiazolidiones Les-6623 and Les-6632

To a stirred solution of 2-(thiazol-2-ylimino)thiazolidin-4-one (comp. 1) or 4-thioxothiazolidin-2-one (comp. 2) (10 mmol) and 4-(3-(dimethylamino)propoxy)benzaldehyde (11 mmol) in ethanol (EtOH) (10 mL) at *ca.* 20 °C, ethylenediamine diacetate (EDDA) (1.8 mg, 10 µmol) was added and the mixture was then heated to reflux for 10 min (for comp. 2) or 2 h (for comp. 1) and left overnight at room temperature. The resultant solid was collected by filtration, washed with methanol (5–10 mL) and diethyl ether, and crystallized from amixture DMF-EtOH (1 : 2).

### 3.2. 5-(4-(3-(Dimethylamino)propoxy)benzylidene)-4-thioxothiazolidin-2-one (Les-6623)

Yield: 73%, mp 245–247 °C. ^1^H NMR (500 MHz, DMSO-d_6_): *δ* 2.07 (t, 2H, *J* = 7.6 Hz, CH_2_), 2.73 (s, 6H, 2*CH_3_), 3.09–3.14 (m, 2H, CH_2_), 4.10 (t, 2H, *J* = 6.0 Hz, CH_2_O), 7.03 (d, 2H, *J* = 8.6 Hz, arom), 7.53 (d, 2H, *J* = 8.6 Hz, arom), 7.89 (s, 1H, =CH). ^13^C NMR (100 MHz, DMSO-*d*_6_): *δ* 24.16 (CH_2_), 42.67 (CH_3_), 54.35 (CH_2_), 64.95 (CH_2_), 115.06, 128.50, 131.27, 133.90, 150.39, 158.60, 163.50 (C=O), 192.2 (C=S). LCMS (ESI): *m*/*z* 323 (98.5%, [M + H]^+^). Anal. Calc. for C_15_H_18_N_2_O_2_S_2_: C 55.88%; H 5.63%; N 19.89%. Found: C 55.60%; H 5.80%; N 19.70%.

### 3.3. 5-(4-(3-(Dimethylamino)propoxy)benzylidene)-2-(thiazol-2-ylimino)thiazolidin-4-one (Les-6632)

Yield: 68%, mp 172–174 °C. ^1^H NMR (500 MHz, DMSO-*d*_6_): *δ* 2.01 (t, 2H, *J* = 6.4 Hz, CH_2_), 2.56 (s, 6H, 2*CH_3_), 2.90 (m, 2H, CH_2_), 4.09 (t, 2H, *J* = 6.2 Hz, CH_2_O), 7.07 (br. s, 2H), 7.38 (br.s, 2H), 7.53 (br.s, 3H). ^13^C NMR (100 MHz, DMSO-d_6_): *δ* 25.04 (CH_2_), 43.47 (CH_3_), 54.76 (CH_2_), 65.37 (OCH_2_), 115.12, 132.28, 133.10, 134.00, 136.79, 137.04, 157.90, 159.58, 167.94, 170.40 (C=O). LCMS (ESI): *m*/*z* 389 (97.8%, [M + H]^+^). Anal. Calc. for C_18_H_20_N_4_O_2_S_2_: C 55.65%; H 5.19%; N 14.42%. Found: C 55.50%; H 5.00%; N 14.60%.

### 3.4. General procedure of synthesis of nanoparticles

Gold nanoparticles AuNPs-PEG-COOH (AuNPs, 10 nm, carboxylic acid functionalized, PEG 3000, Sigma-Aldrich, cat. no. 765457-1ML, OD50, water solution) were used as the nanocarrier platform. The concentration of gold particles provided by the manufacturer was 2.99 × 10^14^ particles per mL. The particles used for drug modification were used as received from the manufacturer without further modification.

Based on the experimentally determined concentration of accessible surface carboxyl groups (1.21 mmol L^−1^) and the manufacturer-reported nanoparticle concentration (2.99 × 10^14^ particles mL^−1^), the number of accessible terminal carboxyl groups was estimated to be approximately 2.4 × 10^3^ groups per nanoparticle. Assuming one terminal carboxyl group per PEG chain and a nanoparticle diameter of 10 nm, the corresponding density of accessible PEG-COOH ligands was estimated to be approximately 7.8 molecules nm^−2^. This calculation should be regarded as an approximation because it reflects the density of accessible titratable terminal groups rather than a direct determination of PEG grafting density.

Compounds Les-6623 and Les-6632 were synthesized according to previously described methods and purified prior to use (^13^; Fig. 1, compounds 3 and 4). Non-covalent electrostatic interactions of AuNPs-PEG-COOH with these ligands were formed between the carboxyl groups of the PEG coating on the nanoparticles and the protonated amino groups present in compounds Les-6623 and Les-6632. For this purpose, aqueous suspensions of AuNPs-PEG-COOH (100 µL) were mixed with 10 µL of ligand solutions in DMSO (*C* = 12.1 mmol dm^−3^) under gentle stirring at room temperature for 2 h.

Initially, the number of active carboxylic groups available for binding with the amine groups present in Les-6623 and Les-6632 was determined. The quantity of functional groups was measured *via* pH-metric titration. Due to the presence of signals from bound carboxylic groups in the form of salts in the bare Au-PEG-COOH nanoparticles (FT-IR spectra, signal at 1636 cm^−1^), an excess of standardized 1 M hydrochloric acid was added to the nanoparticle solution until the pH stabilized. Subsequently, the solution was titrated with a standardized 0.1 M NaOH solution until the pH reached stability.

The concentration of free functional groups was calculated from the second inflection point of the titration curve. The first inflection point corresponded to the back-titration of the excess HCl, while the second indicated the titration of the free carboxylic groups. The titration was performed in triplicate, and the results were averaged, yielding a free carboxylic group concentration of 1.21 mmol dm^−3^.

The amount of drug added (as a solution in DMSO) was calculated based on a 1 : 1 molar ratio. Changes consistent with interaction between the compounds and the carboxyl-containing PEG layer were observed by FT-IR spectroscopy; the disappearance of the C=O signal at 1738 cm^−1^ was observed, alongside the emergence of a signal at 1648 cm^−1^, consistent with formation of an ionic association between the protonated amine groups of the compounds and carboxylate groups at the nanoparticle surface. Quantitative loading efficiency was not directly determined by an orthogonal analytical method therefore, the calculated amount represents the nominal amount introduced during nanoparticle preparation and should not be interpreted as a quantitative mass balance of nanoparticle-associated compound. Changes were also recorded in the fluorescence spectra, where a quenching of fluorescence was observed due to the surface modification of the nanoparticles.

Because particle aggregation was observed during centrifugation or solvent evaporation, no purification step was applied to remove potentially unbound compounds. Consequently, the total amount of compound introduced into the formulation was known, but the fraction remaining associated with the nanoparticles was not quantitatively determined.

### 3.5. Physicochemical characterisation (UV-vis, fluorimetry, DLS)

Zeta potential and particle size distribution were assessed by Dynamic Light Scattering (DLS) with a Zetasizer Ultra system (Malvern Panalytical Ltd, Malvern, UK). Measurements were processed using ZS XPLORER software (Malvern Panalytical Ltd, Malvern, UK) under controlled conditions at 25 °C. The nanoparticles' solutions were diluted in water (5 times, *C* = 5.98 × 10^13^ particles per mL), and the particle size values reported correspond to the mean hydrodynamic diameter derived from five independent measurements. Zeta potential was measured in two attempts each containing 5 independent measurements, the average Zeta potential was then calculated. UV-Vis spectra of diluted water solutions (100 times, *C* = 2.99 × 10^12^ particles per mL) were collected at Jasco V-670 Spectrophotometer (wavelength range 200–800 nm) with water as a reference and Fluorescence measurements of diluted water solutions (100 times, *C* = 2.99 × 10^12^ particles per mL) were performed on Jasco Spectrofluorimeter FP-8500 (JASCO Corporation, Tokyo, Japan). Attenuated Total Reflectance Fourier Transform Infrared (ATR-FTIR) spectra were obtained with an ATR accessory mounted on a Thermo Scientific Nicolet 6700 spectrometer. The spectra were measured from thin sample films. The small drop of nanoparticles' solution was placed on diamond crystal and left for solvent evaporation. Spectral data were recorded in the range of 4000–500 cm^−1^. The aggregation state, size, and morphology of the gold nanoparticles were examined using Transmission Electron Microscopy (TEM) with a Tecnai G2 X-TWIN instrument. For TEM analysis, the samples were deposited onto carbon-coated copper grids.

### 3.6. Docking studies

The molecular docking studies of the synthesized compounds were performed utilizing the Schrödinger suit (2024-4 release). The structure of Dynamin-2 was obtained from the Protein Data Bank (PDB: AF_AFP50570F1) and prepared for further research by the Protein Preparation Wizard of Schrödinger suit for grid generation and molecular docking. The protein was adjusted by removing water molecules and ligands from the PDB file. Bond orders were assigned, and pKa values were generated at a pH of 7 ± 2. Further optimization of the protein was achieved through energy minimization using an OPLS4 force field, resulting in a stable structure suitable for subsequent studies. Next, the SiteMap function of Schrodinger suite was utilized to generate 5 docking sites with the highest possibility of being a potential binding pocket of the chosen protein. Grid generation was performed on the site with the most promising results of site score and drug score. The LigPrep panel of Schrodinger's suit was applied for ligand preparation. Optimized potential for liquid simulation (OPLS4) was applied for energy minimization. Torsional flexibility was given to obtain a better complementary pose. Next, molecular docking was performed using the Glide SP docking protocol of Schrödinger suite. The obtained results are displayed as docking score and ligand efficiency score in kcal mol^−1^. Visualization of the molecular docking results was performed using the Maestro interface of the Schrödinger suit.

### 3.7. Cell culture

The human neuroblastoma cell line SH-SY5Y (ATCC, CRL-2266, LOT: 70047955) was used to evaluate the efficacy of nanoparticles conjugated with endocytosis inhibitors in case of HIV-1 CPP Tat, while the human lung carcinoma epithelial cell line A549 (ATCC, CCL-185™) and the normal human bronchial epithelial cell line BEAS-2B (ATCC, CRL-3588) were employed to assess the inhibitory potential of novel derivatives against SARS-CoV-2 spike protein internalization. SH-SY5Y cells were maintained in DMEM/F-12 medium (Gibco, New York, NY) supplemented with 10% fetal bovine serum (FBS; Gibco, New York, NY) and 1% Penicillin-Streptomycin (Corning, Kennebunk, ME), whereas A549 and BEAS-2B cells were cultured in DMEM (Gibco, New York, NY) containing 10% FBS and 1% Penicillin-Streptomycin under standard conditions. In addition, glioblastoma cell lines A-172 (ATCC, CRL-1620) and U-87 MG (ATCC, HTB-14), as well as the pancreatic carcinoma cell line PANC-1 (ATCC, CRL-1469), were included to broaden the cellular models of cytotoxicity. Cells were cultured in cell culture flasks (Sarstedt, Numbrecht, Germany) at 37 °C, in an atmosphere containing 5% CO_2_, until they reached 90–95% subconfluence. For experiments, cells were then washed with phosphate buffered saline without calcium and magnesium (PBS; Corning, Kennebunk, ME, USA) and treated with 0.05% trypsin with 0.02% EDTA (Corning, Kennebunk, ME, USA). Cells were counted using a Scepter 3.0 cell counter (Merck Millipore, Burlington, MA, USA) prior to seeding into plates.

### 3.8. Cytotoxicity evaluation

The cytotoxic activity of compounds was evaluated *in vitro* using human cell lines. Cells were seeded into 96-well plates at density 1 × 10^4^ per well and allowed to adhere for 24 h. The cells were then treated with the tested compound or nanoparticles at final concentrations of 3.125, 6.25, 12.5, 25.0, and 100.0 µM for 48 h. Cell viability was determined using MTT assay. Briefly, an MTT (3-(4,5-dimethyl-2-thiazolyl)-2,5-diphenyl-2*H*-tetrazolium bromide; Sigma-Aldrich, St. Louis, MO) stock solution (5 mg mL^−1^) in phosphate-buffered saline (PBS; Corning, Kennebunk, ME) was added to each well, and the plates were incubated for 2 h at 37 °C to allow the formation of formazan crystals. Subsequently, the culture medium was removed, and the formazan crystals were dissolved in 100 µL of lysis buffer (pH 4.7) containing 5% SDS, 3% *N*,*N*-dimethylmethanamide (DMF), 2% acetic acid, 25 mM HCl. The absorbance was measured at 570 nm using an Absorbance 96 microplate reader (Byonoy GmbH, Hamburg, Germany). Cell viability was expressed as a percentage relative to untreated control cells.

### 3.9. Analysis of endocytosis inhibition

Cells were seeded on sterile coverslips at density 1 × 10^5^ per well for 24 h in conditions previously described in section Cell culture. The cells were pretreated or not for 1 h with the free compounds Les-6623 and Les-6632 at a final concentration of 10 µM or with AuNPs_Les-6623 and AuNPs_Les-6632 corresponding to a nominal inhibitor concentration of 10 µM, calculated from the amount of inhibitor introduced during nanoparticle formulation. Because the actual loading efficiency was not directly quantified, this value represents the nominal inhibitor concentration used for dosing rather than a directly measured concentration of nanoparticle-associated inhibitor. Chlorpromazine was used at a final concentration of 30 µM. Then, either 25 µM of Transferrin Alexa Fluor 647 conjugate (#T23366, Thermo Fisher Scientific, Eugene, OR) 1 µM Tat CPP-Cy5 (sb-PEPTIDE, Saint Egrève, France) or 1 µM SARS-CoV-2 Spike protein labelled with Alexa 647 (#AFR10796; R&D Systems, Minneapolis, MN, USA) were added to the cell medium for 30 min, followed by cell wash, and fixation with 4% paraformaldehyde. After incubation, 10 µg mL^−1^ of 4′,6-diamidino-2-phenylindole (DAPI) (Sigma-Aldrich, St. Louis, MO) was added for 3 min at room temperature. Observations were made with SP8 Leica (Leica Microsystems, GmbH, Wetzlar, Germany). Results were analyzed using ImageJ software (Bethesda, MD), where the fluorescence intensity of the fluorescently-labelled cargo was evaluated relative to the control and reference compound.

In parallel, quantitative analysis of transferrin cellular uptake was also performed by flow cytometry (FACS). Cells were seeded on 12-well plates at density 1 × 10^5^ per well for 24 h in conditions previously described in section Cell culture. The cells were pretreated or not for 1 h with tested compounds and nanoparticles in final concentration 5 and 10 µM, chlorpromazine at 30 µM. Then 25 µM of Transferrin Alexa Fluor 647 conjugate (#T23366, Thermo Fisher Scientific, Eugene, OR) were added to the cell medium for 30 min. Then samples were immediately analyzed using a BD FACSCanto II flow cytometer (Becton Dickinson Biosciences, San Jose, CA, USA), with 20 000 events recorded per sample. The results were then evaluated using FACSDiva software version 6.1.3 (BD Biosciences Systems, San Jose, CA, USA).

## 4. Conclusions

In this study, we demonstrated that gold nanoparticles functionalized with Les-6623 and Les-6632 reduce uptake of tested fluorescent cargoes. The nanoparticle formulations reduced cellular fluorescence associated with transferrin, HIV-1 CPP Tat, and SARS-CoV-2 Spike protein under the experimental conditions tested. Free chlorpromazine was included as a reference inhibitor, and differences in activity were observed between the formulations. However, because the amount of nanoparticle-associated inhibitor was not quantitatively determined and the comparison with chlorpromazine was not format-matched, the present data do not establish that the nanoparticle format itself is responsible for the observed differences in activity. These findings provide a basis for further studies using quantitative drug-loading measurements and format-matched controls to determine the contribution of nanoparticle presentation to the biological activity of these inhibitors.

## Conflicts of interest

There are no conflicts to declare.

## Abbreviations

^1^H NMR: proton nuclear magnetic resonance
^13^C NMR: carbon-13 nuclear magnetic resonance
A172: human glioblastoma cell line
A549: human lung carcinoma cell line
ACE2 receptor: angiotensin-converting enzyme 2
ATR-FTIR: attenuated total reflectance – Fourier transform infrared spectroscopy
BEAS-2B: human bronchial epithelial cell line
DLS: dynamic light scattering
DDR: DNA damage response
GTPase: guanosine triphosphatase
HIV: human immunodeficiency virus
HIV-1 Tat: trans-activator of transcription from HIV-1 virus
HIV-1 CPP Tat: HIV-1 cell-penetrating peptide Tat
LC-MS: liquid chromatography – mass spectrometry
MCF-7: human breast adenocarcinoma cell line
MTT: 3-(4,5-dimethylthiazol-2-yl)-2,5-diphenyltetrazolium bromide assay
Panc-1: human pancreatic carcinoma cell line
PEG: polyethylene glycol
SARS-CoV-2: severe acute respiratory syndrome coronavirus 2
SH-SY5Y: human neuroblastoma cell line
TEM: transmission electron microscopy
U87-MG: human glioblastoma cell line
UV–Vis: ultraviolet–visible spectroscopy

## Supplementary Material

RA-OLF-D6RA07148A-s001

## Data Availability

The data supporting this article have been included as part of the supplementary information (SI). Supplementary information is available. See DOI: https://doi.org/10.1039/d6ra07148a.
