# Supplementary material for: Nanoparticles improve targeting and efficacy of endocytosis inhibitors
Source: RSC Adv. 2026 Oct 22;16(48):65271–83. doi: 10.1039/d6ra07148a (PMC13688105; doi:10.1039/d6ra07148a)
Supplement: Supplementary file 1 [file RA-016-D6RA07148A-s001.pdf]

This journal is © The Royal Society of Chemistry

1. Supplementary data

7.1. The spectral data of compounds Les-6623 and Les-6632

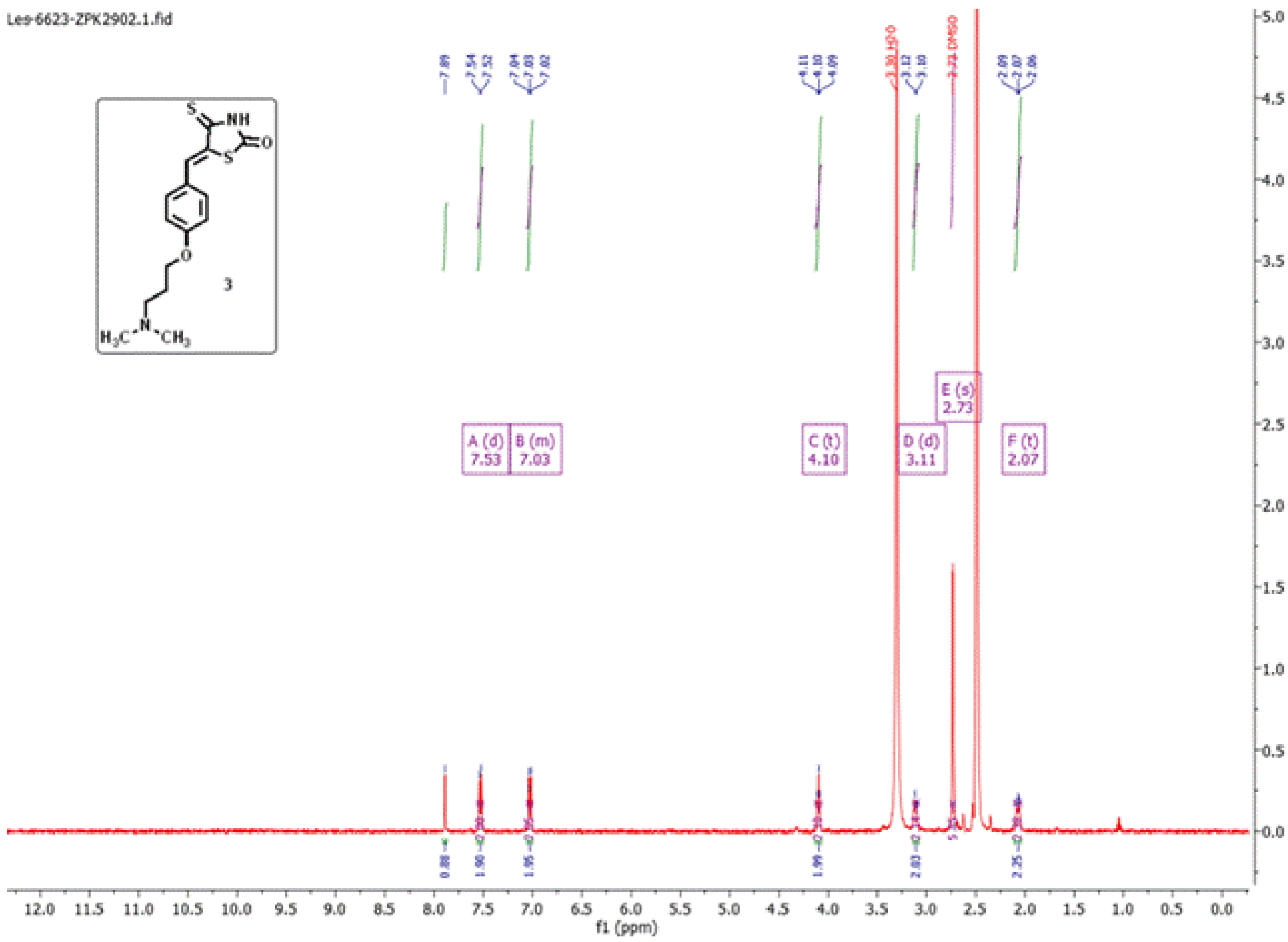

Figure S1. ^{1}□ NMR spectrum of compound **Les-6623** (3).

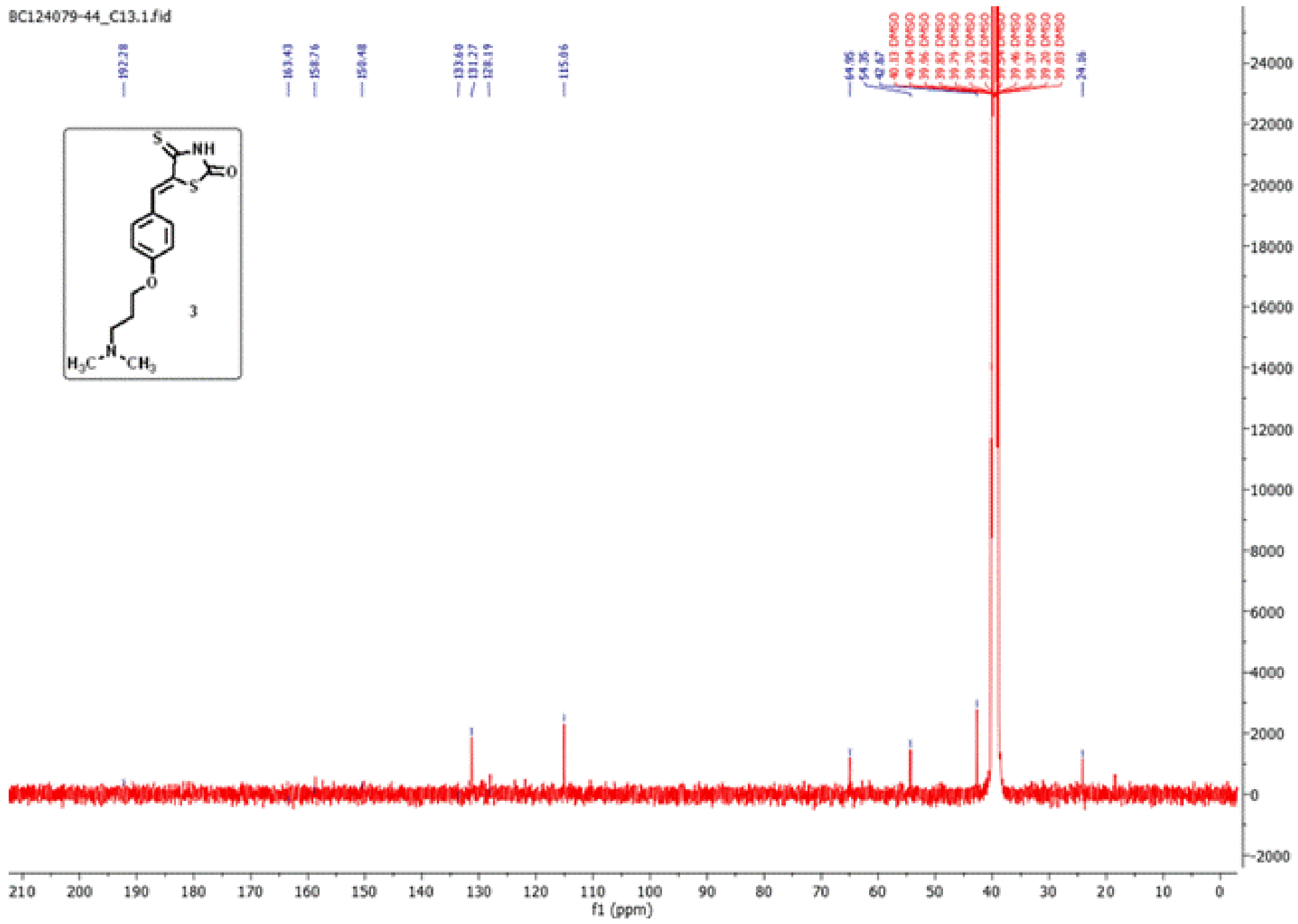

Figure S2. ^{13}C NMR spectrum of compound **Les-6623** (3).

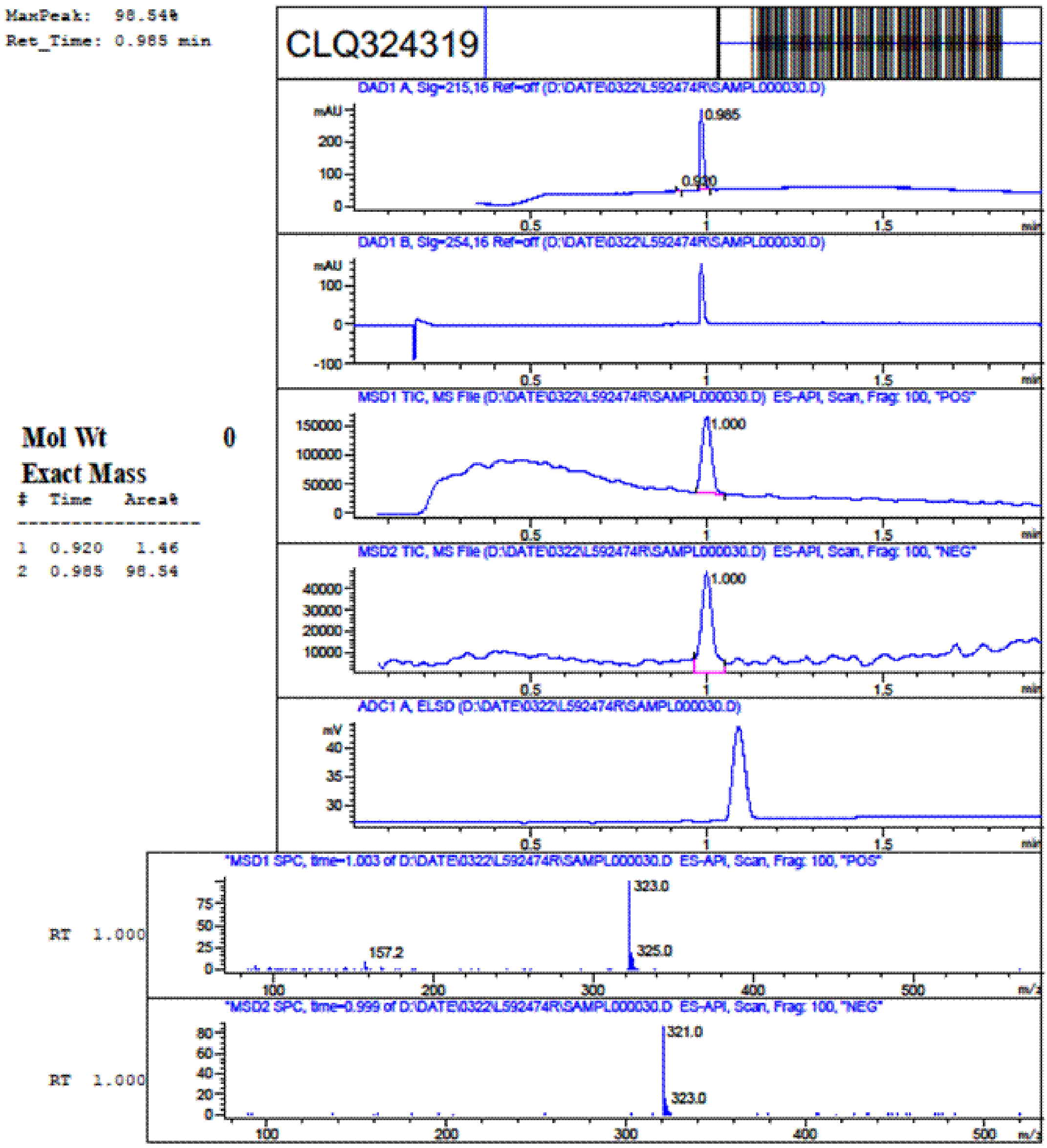

Figure S3. LC-MS spectrum of compound **Les-6623** (**3**).

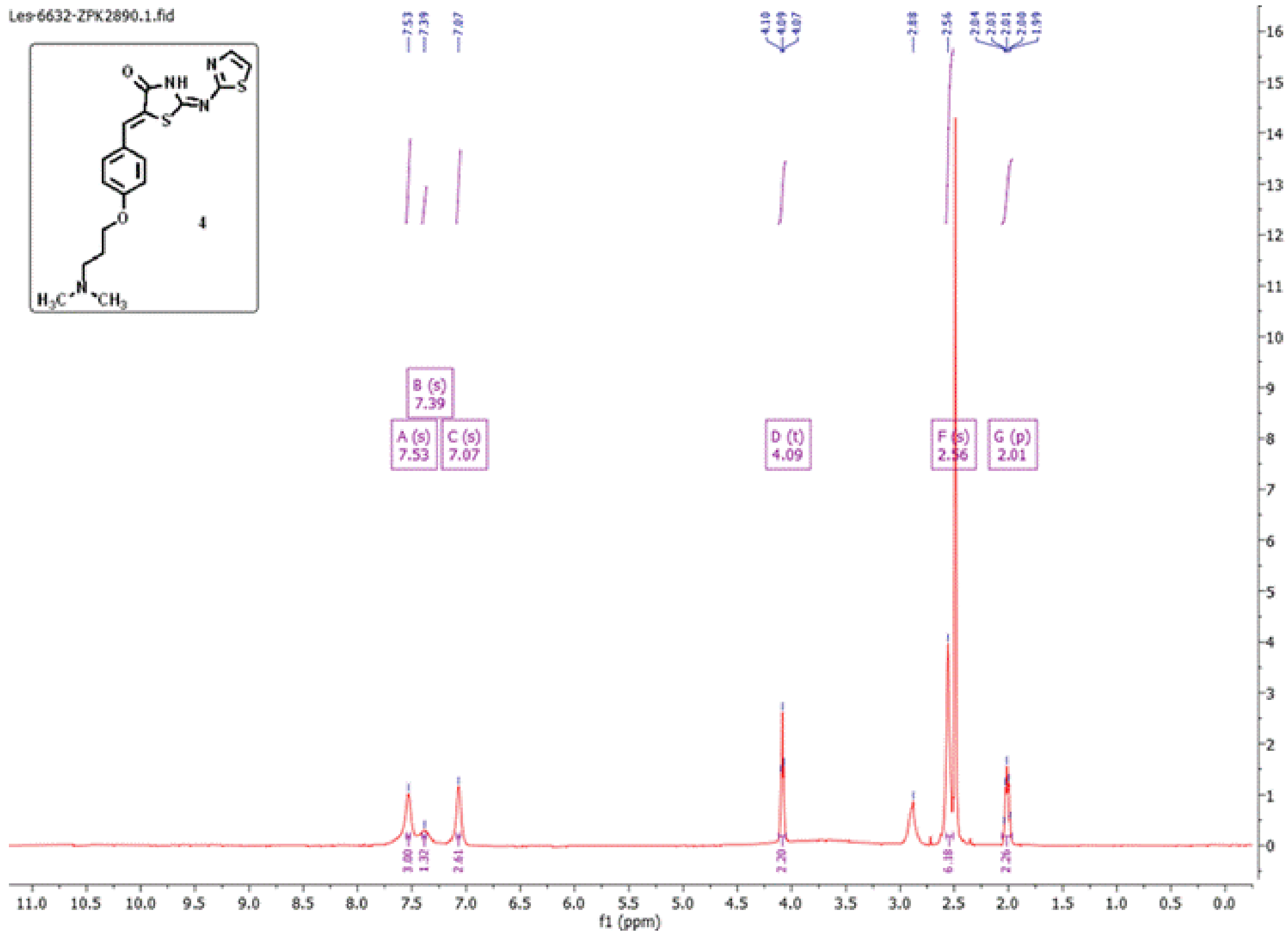

Figure S4. ^{1}H NMR spectrum of compound **Les-6632 (4)**

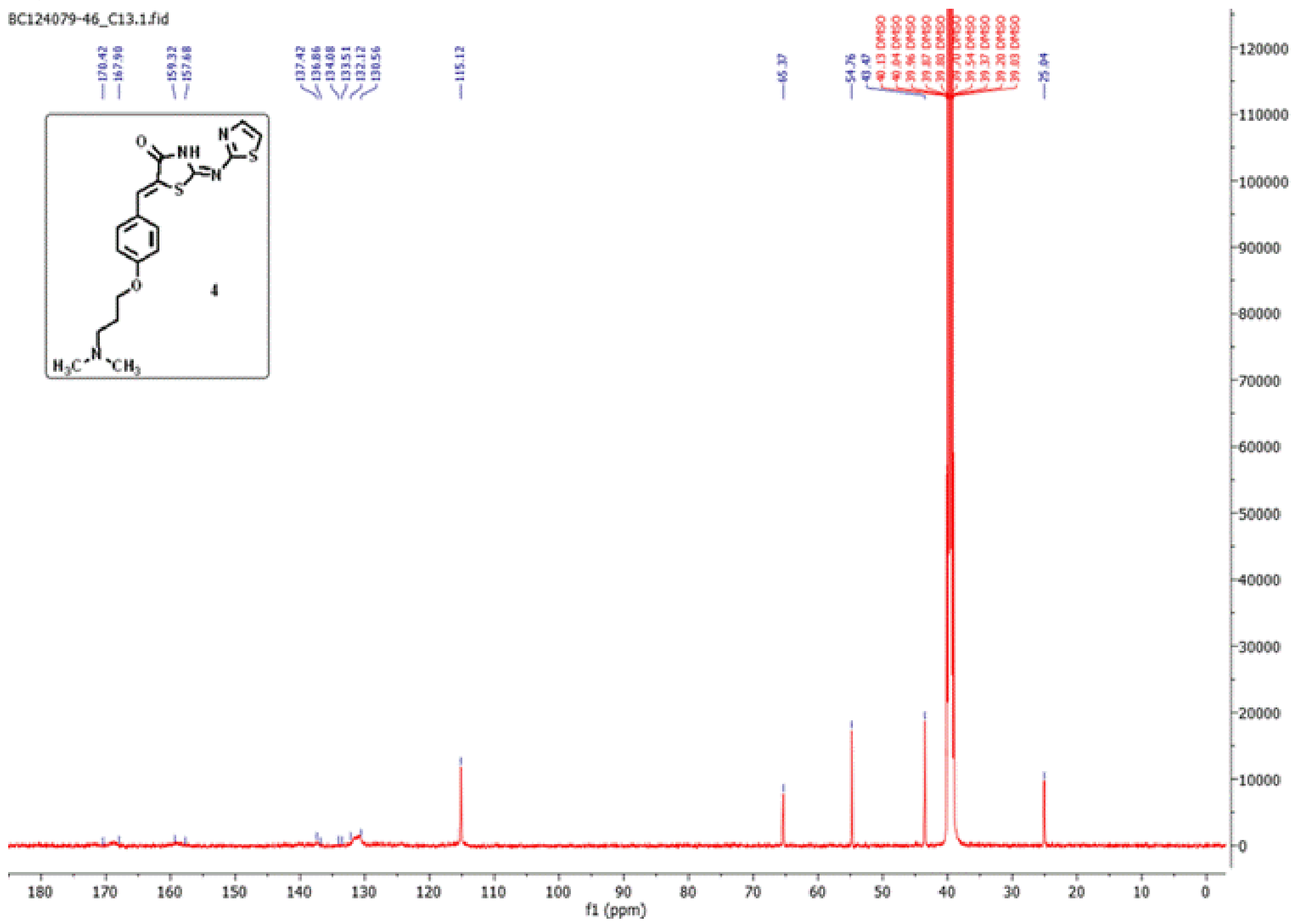

Figure S5. ^{13}C NMR spectrum of compound **Les-6632 (4)**

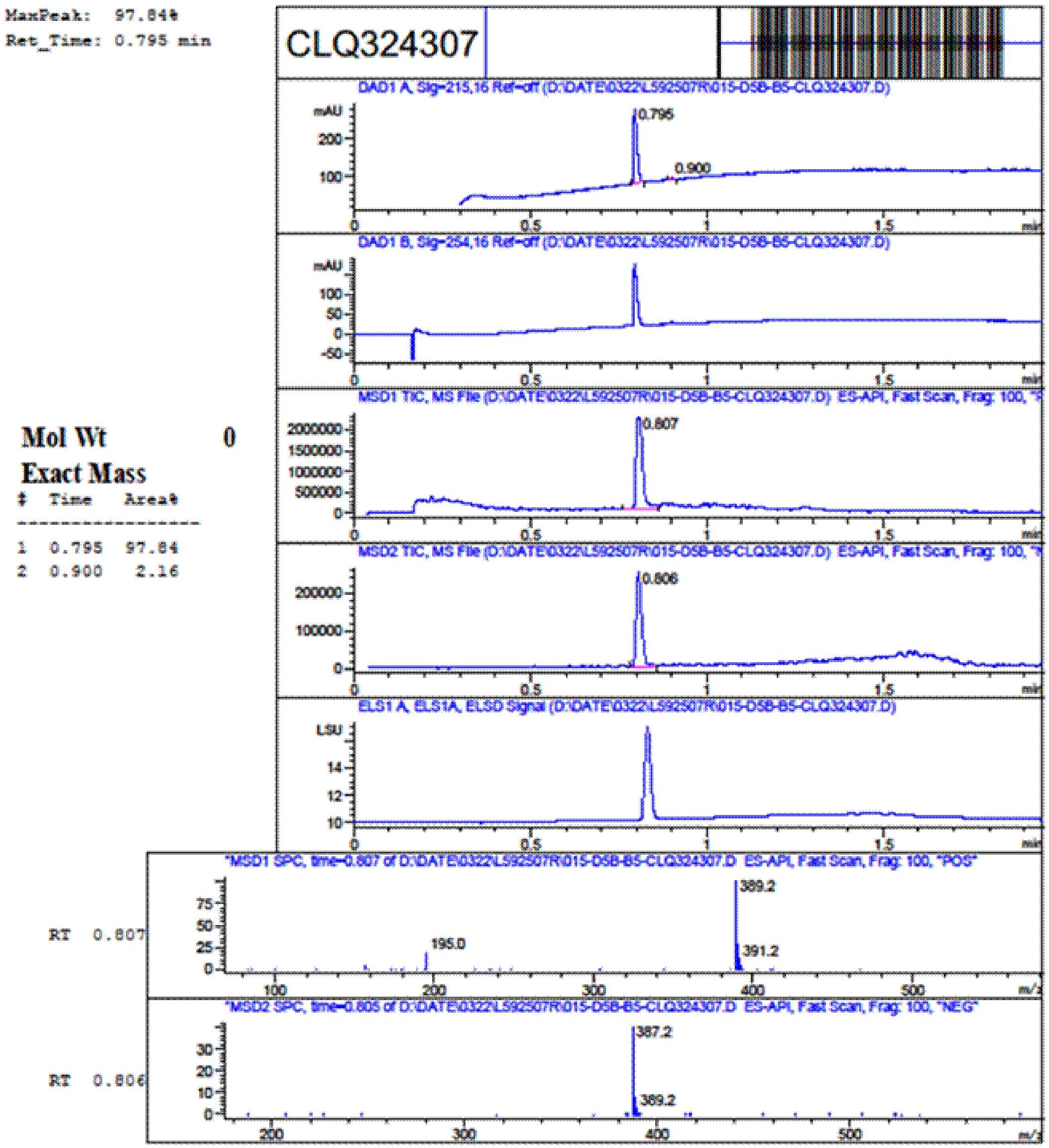

Figure S6. LC-MS spectrum of compound **Les-6632** (**4**)

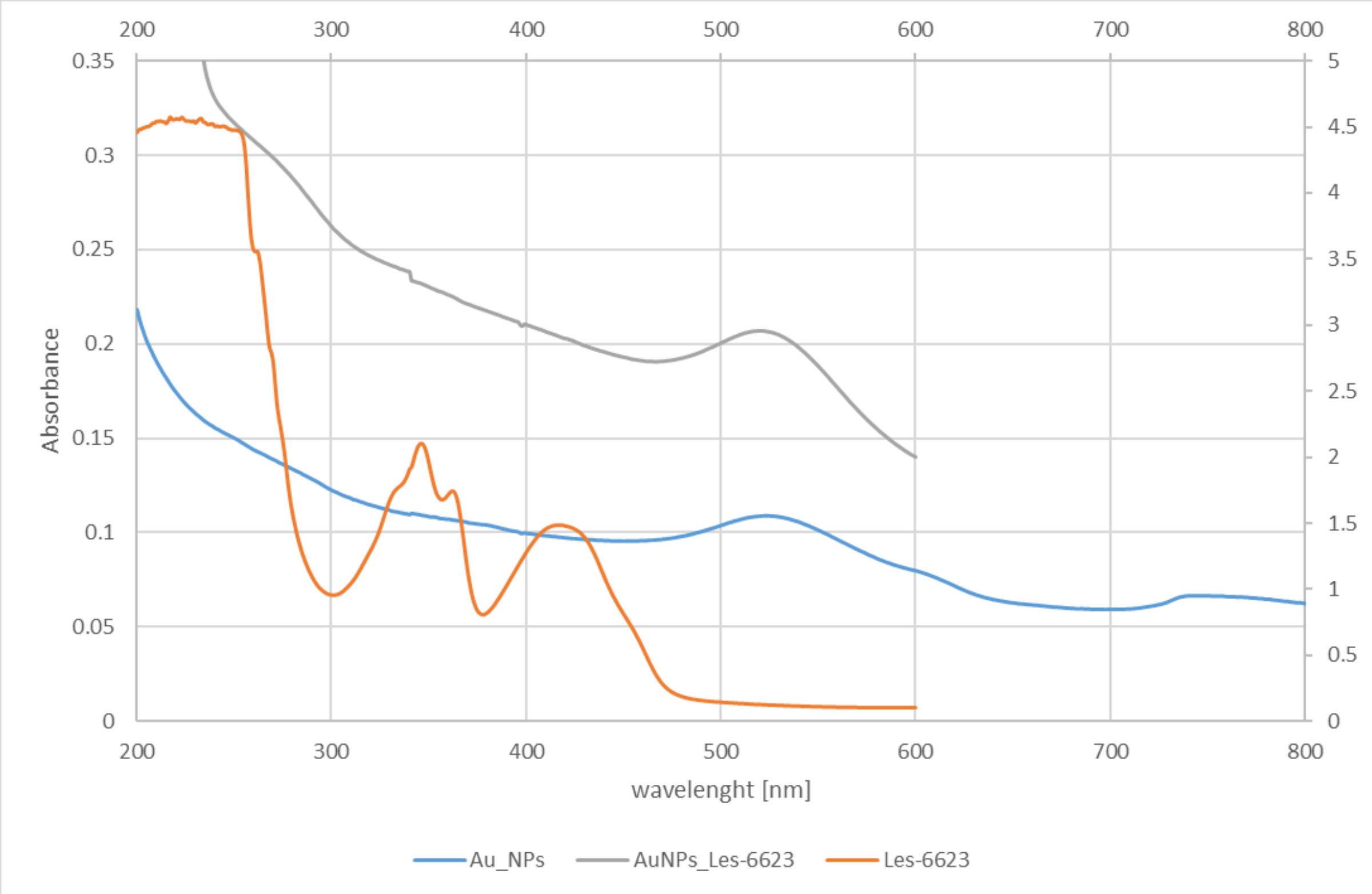

Figure S7. UV-Vis absorption spectra of gold nanoparticles coated with PEG and modified with **Les-6623** compound

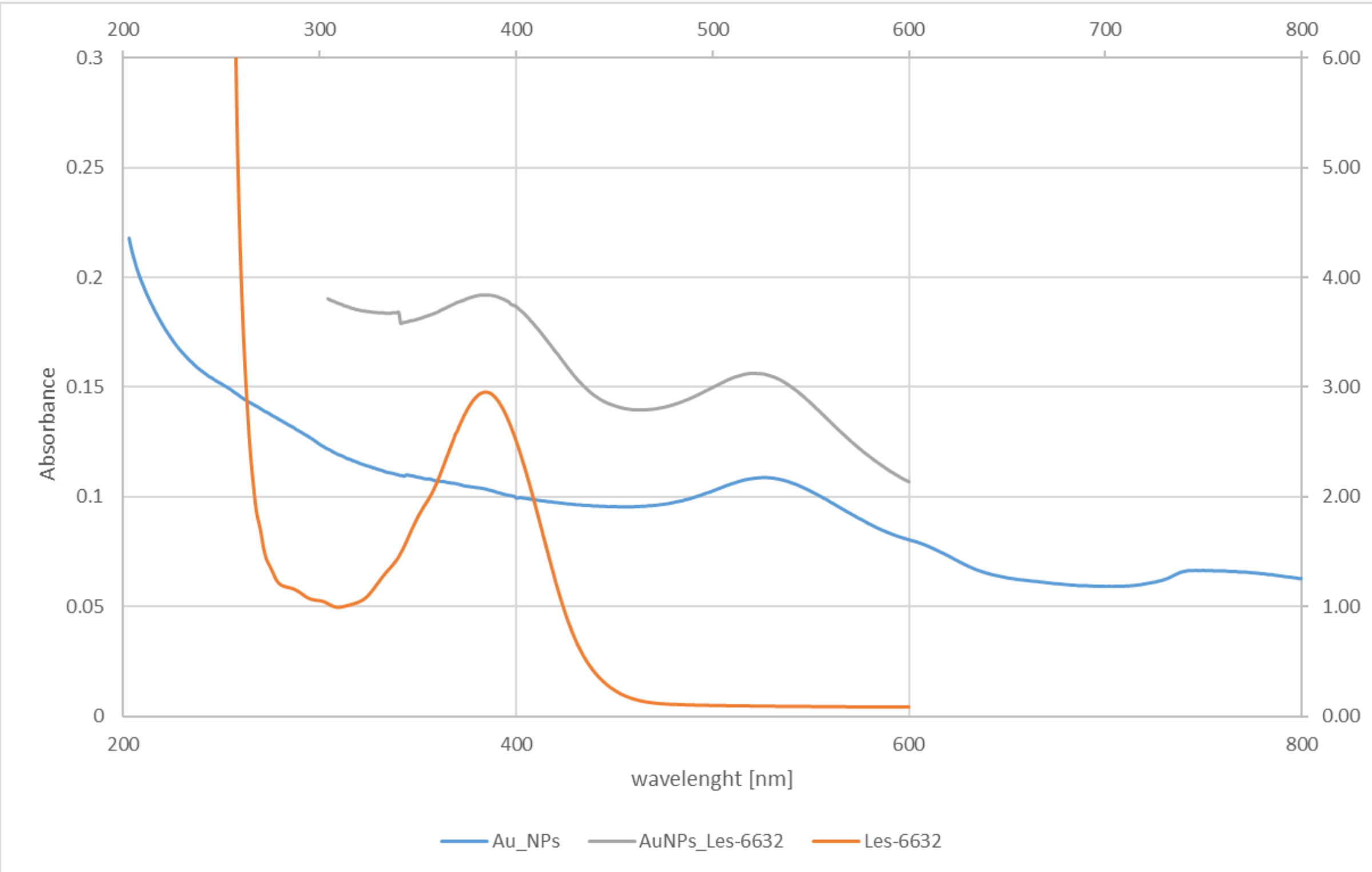

Figure S8. UV-Vis absorption spectra of gold nanoparticles coated with PEG and modified with **Les-6632** compound

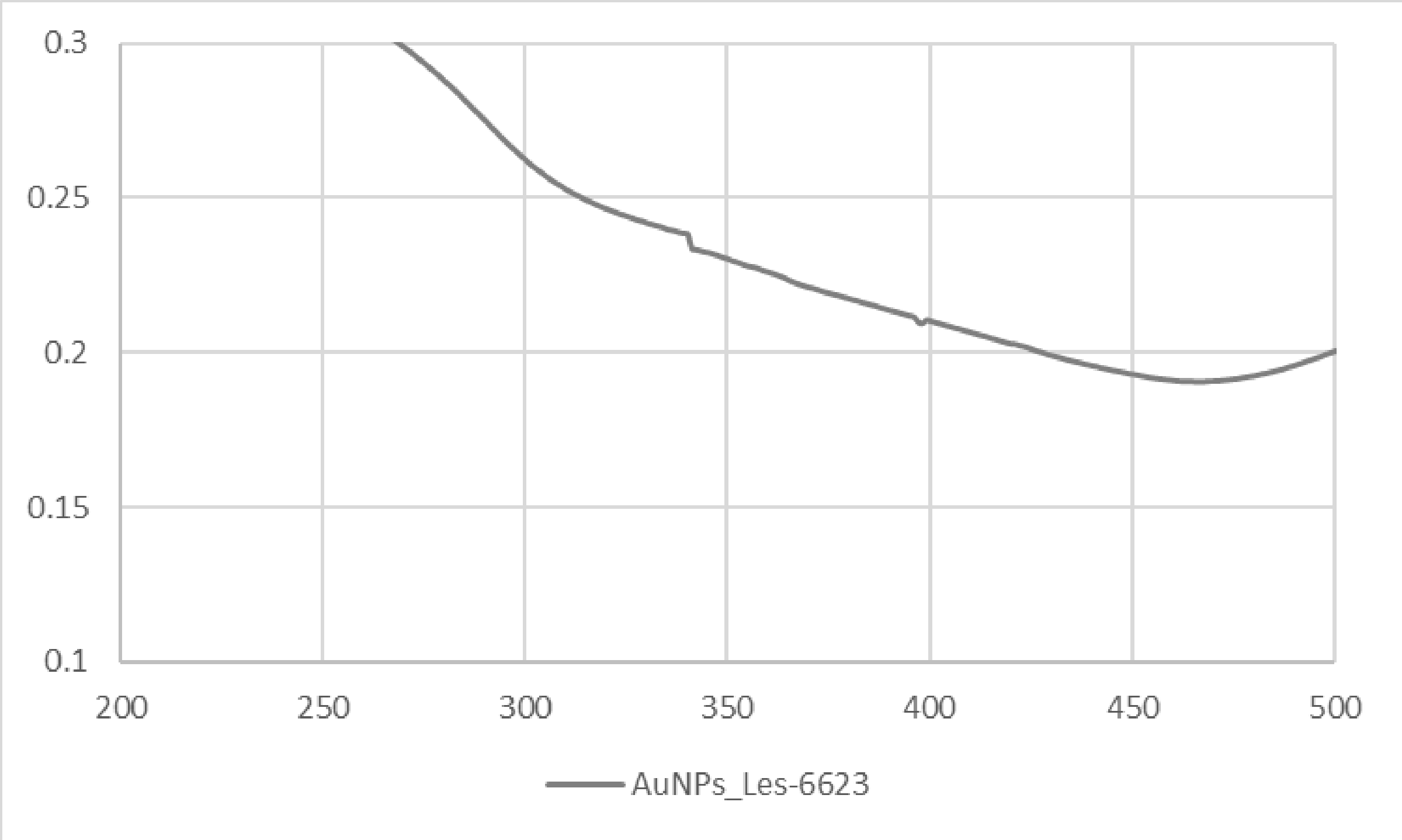

Figure S9. Within the absorption range characteristic of Les-6623, the spectrum of AuNPs_Les-6623 showed a slight bulge, a low-intensity band coinciding with the spectral region where the light source switches from halogen to deuterium lamp, which appears as a small step in the spectrum. The detection of such a signal is hindered by technical limitations

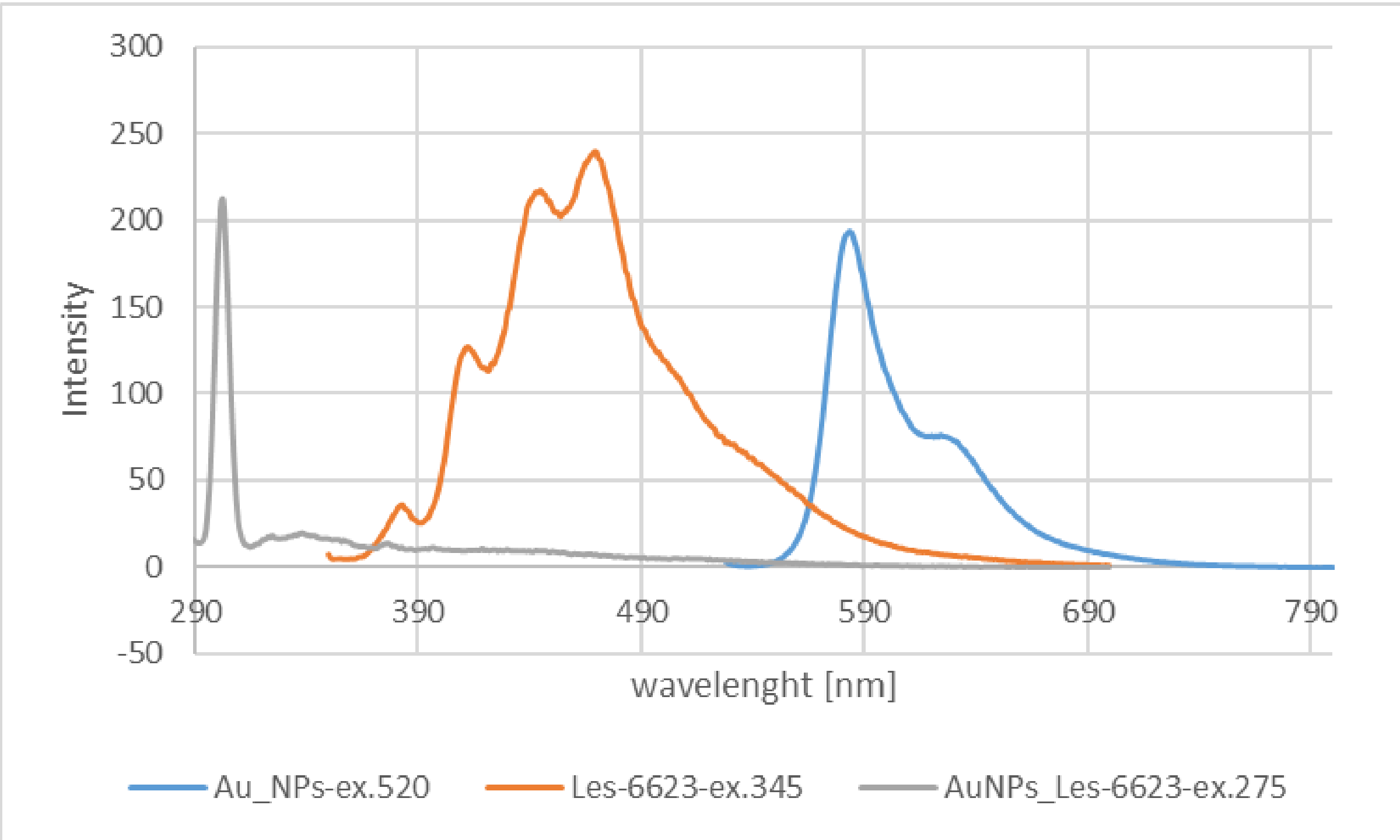

Figure S10. Comparison of spectra of fluorescence gold nanoparticles coated with PEG, modified with Les-6623 compound and compound Les-6623 alone

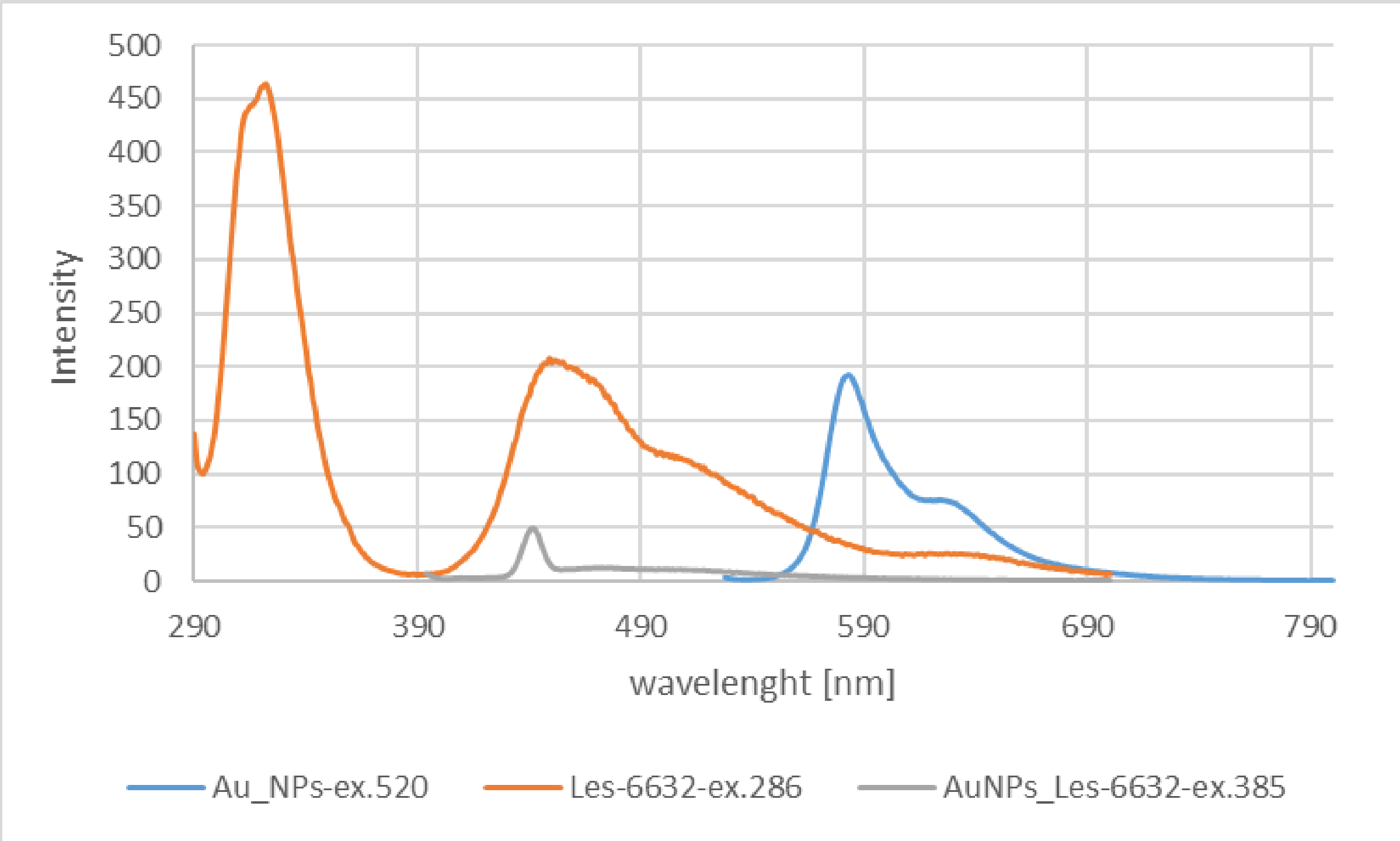

Figure S11. Comparison of spectra of fluorescence gold nanoparticles coated with PEG, modified with Les-6632 compound and compound Les-6632 alone

NPs	ζ	σ	size
AuNPs	-8,306	0,7033	9-42 nm
AuNPs-Les-6623	-22,95	1,199	9-37 nm
AuNPs-Les-6632	-8,303	0,9375	13-44 nm

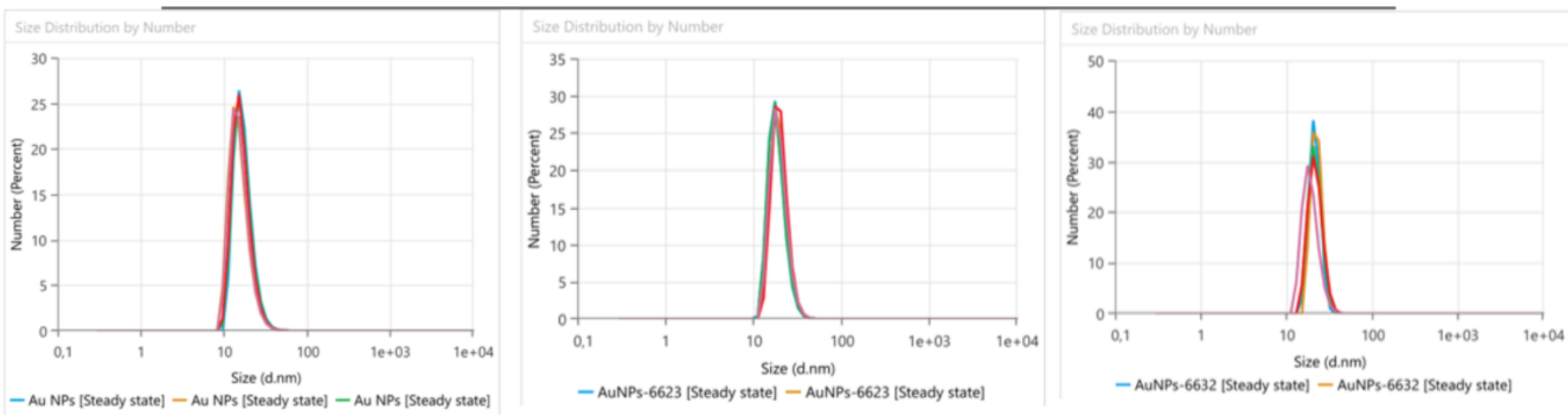

Name (NPs_Au_Les-6623)	Mean 1*	Mean 2*	Name (NPs_Au_Les-6632)	Mean 1*	Mean 2*
Zeta Potential (mV)	-23.023	-21.626	Zeta Potential (mV)	-0.766	-8.752
Conductivity (mS/cm)	0.024	0.022	Conductivity (mS/cm)	0.039	0.055
Wall Zeta Potential (mV)	0	0	Wall Zeta Potential (mV)	0	0
Quality Factor	3.676	4.04	Quality Factor	1.303	2.559
*from 5 measurements			*from 5 measurements		

Figure S12. DLS and zeta potential results from ZetaSizer Ultra

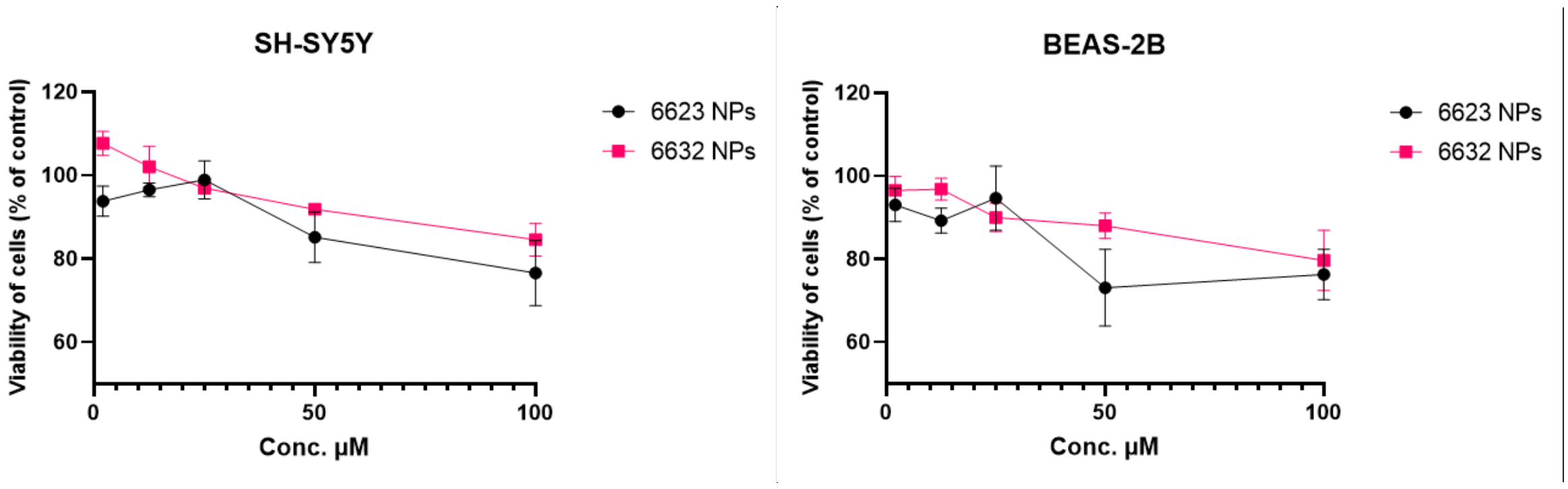

Figure S13. Graphs with cytotoxicity assessed with MTT assay of nanoparticles AuNPs_Les-6623 and AuNPs_Les-6632 after 48h incubation
